# Development of a high-throughput tailored imaging method in zebrafish to understand and treat neuromuscular diseases

**DOI:** 10.3389/fnmol.2022.956582

**Published:** 2022-09-20

**Authors:** Léa Lescouzères, Benoît Bordignon, Pascale Bomont

**Affiliations:** ^1^ERC Team, Institut NeuroMyoGéne-PGNM, Inserm U1315, CNRS UMR 5261, Claude Bernard University Lyon 1, Lyon, France; ^2^Montpellier Ressources Imagerie, BioCampus, CNRS, INSERM, University of Montpellier, Montpellier, France

**Keywords:** zebrafish model, imaging methodology, neuromuscular system, disease mechanisms, drug screening

## Abstract

The zebrafish (*Danio rerio*) is a vertebrate species offering multitude of advantages for the study of conserved biological systems in human and has considerably enriched our knowledge in developmental biology and physiology. Being equally important in medical research, the zebrafish has become a critical tool in the fields of diagnosis, gene discovery, disease modeling, and pharmacology-based therapy. Studies on the zebrafish neuromuscular system allowed for deciphering key molecular pathways in this tissue, and established it as a model of choice to study numerous motor neurons, neuromuscular junctions, and muscle diseases. Starting with the similarities of the zebrafish neuromuscular system with the human system, we review disease models associated with the neuromuscular system to focus on current methodologies employed to study them and outline their caveats. In particular, we put in perspective the necessity to develop standardized and high-resolution methodologies that are necessary to deepen our understanding of not only fundamental signaling pathways in a healthy tissue but also the changes leading to disease phenotype outbreaks, and offer templates for high-content screening strategies. While the development of high-throughput methodologies is underway for motility assays, there is no automated approach to quantify the key molecular cues of the neuromuscular junction. Here, we provide a novel high-throughput imaging methodology in the zebrafish that is standardized, highly resolutive, quantitative, and fit for drug screening. By providing a proof of concept for its robustness in identifying novel molecular players and therapeutic drugs in giant axonal neuropathy (GAN) disease, we foresee that this new tool could be useful for both fundamental and biomedical research.

## Introduction

Over the last 30 years, the zebrafish (*Danio rerio*) has become the model of choice for many fundamental and translational research studies. With about 70% ortholog genes shared with the human genome (Barbazuk, 2000; Howe et al., 2013), the zebrafish is largely used in the fields of embryology, pharmacology, and toxicology to expand our knowledge on complex biological processes and accelerate the emergence of precision medicine (MacRae and Peterson, 2015). It has also become a preferred species for the modeling of various diseases, including cancer, and cardiovascular, metabolic, and neuromuscular diseases (NMDs) (Seth et al., 2013; Patton et al., 2021).

The success of this animal model largely relies on the numerous advantages it offers to the research community. Regarding practical aspects, the husbandry of zebrafish is relatively simple and inexpensive. Its high fertility rate (∼200 embryos per clutch), rapid and external development, and transparent skin allow direct access to all developmental stages from embryo (one-cell zygote at 0 h post-fertilization, hpf, to hatching at 2–3 days post-fertilization, dpf), to larva (from 3 to 29 dpf) and juvenile (until 6 weeks). As a consequence, zebrafish presents many advantages: (i) genetic manipulation can be achieved at an early stage of embryonic development (injection of antisense oligonucleotides/mRNA for transient effects, or CRISPR agents for the creation of stable transgenic lines), (ii) transparency of embryos allows for the visualization of internal structures and networks at the level of the whole organism, (iii) small-size (0.9–3.5 mm) zebrafish embryos can be kept in 96- or 384-well plates, and (iv) chemical compounds can be administered by simply adding to water (Zon and Peterson, 2005). In practice, the study of zebrafish is cost-effective and compatible with large-scale/high-throughput pharmacological screening (Rennekamp and Peterson, 2015), explaining why it has become the leading model organism for chemical screening and drug discovery.

The zebrafish model has particularly proven to be valuable in vertebrate neuromuscular system studies, which have deepened our knowledge in normal axonal/muscle synapse development and degeneration (Guyon et al., 2007; McLean and Fetcho, 2008; Li et al., 2017) and in identifying the deficits underlying neuromuscular pathologies (Panzer et al., 2005, 2006). The locomotion-sustaining human neuronal circuitry, which is highly conserved in zebrafish, was characterized, thanks to this species (Kimmel et al., 1995; Beattie, 2000). In particular, studies using this model contributed to the identification of molecular signals required for the establishment of motor networks (Berg et al., 2018), neuromuscular junction (NMJ) development, NMJ maintenance, synaptogenesis (Jing et al., 2009, 2010; Banerjee et al., 2011), and identification of muscle precursor types (Devoto et al., 1996).

With the ease of genetic manipulation, various transgenic zebrafish lines represent powerful models to study human diseases, in an era where whole-genome/-exon sequencing generates big data of genetic variants in patients (Pipis et al., 2019; Westra et al., 2019; Herman et al., 2021). In this regard, the zebrafish can be extremely valuable in (1) discovering the disease gene among potential candidates, (2) identifying pathological variants of a known gene, and (3) deciphering pathological mechanisms (McCammon and Sive, 2015; Vaz et al., 2019).

Here, we first describe the key events in zebrafish neuromuscular system development and its resemblance to human counterpart. We then briefly present how the creation of disease models of the neuromuscular system contributes to the understanding of this specific synapse in health, as well as the underlying mechanisms in disease. We then focus on the current behavioral and imaging-based techniques used in the zebrafish, putting in perspective the need to generate tools with greater precision to enable a deeper exploration of the neuromuscular system and to apprehend specificity in disease, an essential aspect for personalized medicine. Finally, we present a novel quantitative and standardized imaging methodology with a proof of concept for its effectiveness in identifying both novel regulators of the neuromuscular system and therapeutic drugs using high-throughput screening.

## Zebrafish is a model of choice to study the motor system in human

**FIGURE 1:**
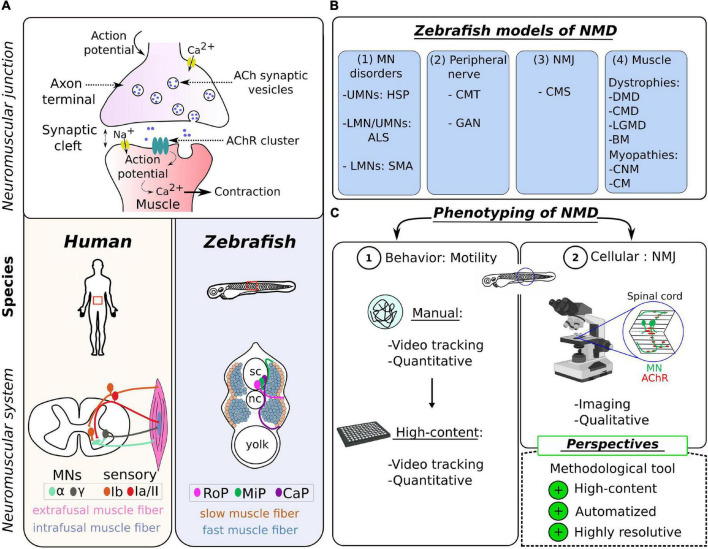
Zebrafish as a model of choice to study the neuromuscular system in human. **(A)** Schematic representation of the neuromuscular synapse (top) and the topographical differences (bottom) between the zebrafish and the human species. **(B)** Table summarizing the different NMD pathologies modeled in zebrafish, categorized into four groups according to the first disease target (see Table 1 for details). UMN, upper motor neuron; LMN, lower motor neuron; HSP, hereditary spastic paraplegia; ALS, amyotrophic lateral sclerosis; SMA, spinal muscular atrophy; CMT, Charcot–Marie–Tooth; GAN, giant axonal neuropathy; NMJ, neuromuscular junction; CMS, congenital myasthenic syndrome; DMD, Duchenne muscular dystrophy; CMD, congenital muscular dystrophy; LGMD, limb–girdle muscular dystrophy; BM, Bethlem myopathy; CNM, centronuclear myopathies; CM, congenital myopathy. **(C)** Scheme summarizing the common tools for NMD phenotyping in the zebrafish, divided into two levels of investigation: (1) the locomotion at the behavioral level and (2) the visualization of the NMJ at the cellular level; and highlighting the methodological challenge presented as perspectives in this review.

At the cellular level, the NMJ is made up of the same components across species (Figure 1A). It is formed by two entities: the presynaptic motor neuron (MN) of the nervous system and the postsynaptic muscle fiber, which are separated by a synaptic cleft. The transmission of electrical input along the MN axon is achieved by a spread of action potentials. Upon activation, the fusion of vesicles in MN terminals results in a release of acetylcholine (ACh) into the synaptic cleft, which activates postsynaptic nicotinic ACh receptors (AChRs) in the postsynaptic muscle fibers (Kummer et al., 2006). This event depolarizes the muscle cell and triggers calcium release from the sarcoplasmic reticulum to initiate muscle contraction. Many molecular, histological, and ultrastructural features of NMJ development and integrity are well conserved between mammals and zebrafish (Swenarchuk, 2019). Among other examples, the key role of AChR pre-patterning in guiding MN terminals to the muscle fibers was discovered in the zebrafish model (Panzer et al., 2005), as well as the role of the Wnt pathway in this process (Jing et al., 2010).

The skeletal muscle, which is the only muscle tissue voluntary controlled by the central nervous system, is closely related in zebrafish and humans, at histological, biochemical, and ultrastructural levels (Luna et al., 2015). These similarities include the preservation of two sets of MNs, two main types of muscle fibers (slow and fast), the dystrophin-associated protein (DAPC) complex, and the excitation–contraction coupling system (Berger and Currie, 2012). Differences are restricted to morphological variables such as MN axon diameters and average area of AChR clusters (Jones et al., 2017; Boehm et al., 2020), and the specificity/subclassification of muscle fibers, which have their source in an adaptation of locomotor behavior. In humans, there are two major types of spinal MNs located in the anterior horn of the spinal cord, differentiated according to the size of the soma and their innervation (Figure 1A): α-MNs and γ-MNs (Kanning et al., 2010; Manuel and Zytnicki, 2011). The α-MNs are the most abundant type of MNs in mammals. They innervate extrafusal muscle fibers classified into different subtypes, whose contractile properties differ: slow-twitch, fatigue-resistant (S), and fast-twitch fibers, which are further subdivided into fatigue-resistant (FR) and fatigable (FF) fibers (Brooke and Kaiser, 1970; Burke et al., 1971). The γ-MNs innervate intrafusal muscle fibers responsible for muscle tone and proprioception. Zebrafish spinal MNs are also divided into two groups, differentiated in two distinct waves during development: primary motor neurons (pMNs) and secondary motor neurons (sMNs) innervating specific musculature (Myers, 1985; Eisen et al., 1986). There is also a subclassification of primary motor neurons (pMNs) in each hemisegment, originally designated according to their specific location as caudal primary motor neuron (CaP); middle primary motor neuron (MiP), and rostral primary motor neuron (RoP) (Figure 1A), innervating different muscle fiber territories. Motor neurons from the second wave (sMNs) are considered to be equivalent to α-MNs in humans (Beattie et al., 1997). They have small-size somas, are more numerous, and innervate both deeper fast-twitch muscle and superficial slow-twitch muscle fibers (Menelaou and McLean, 2012; Bello-Rojas et al., 2019). It is worth noting that the proportion of fast muscles is much higher in zebrafish than in mammals, an adaptative response to the need to produce bursting and rapid powerful movements.

## Zebrafish mimics pathologies of the neuromuscular system

The genome is highly conserved between the human and zebrafish species, with a homology for over 80% of disease-causing genes (Howe et al., 2013). This evolutionary characteristic fostered the generation of zebrafish mutants to model pathologies of the human neuromuscular system (Pappalardo et al., 2013). Strategies of transient genetic manipulations using microinjection (Eisen and Smith, 2008) (gene inactivation to model recessive diseases or mRNA overexpression for dominant pathologies), followed by genome editing (Gaj et al., 2013; Schmid and Haass, 2013), have been successfully used to assess the significance of pathological mutations associated with neurodevelopmental and neurodegenerative conditions, including NMDs.

Here, NMDs are defined as pathologies inducing muscle weakness and atrophy (Efthymiou et al., 2016). Thus, NMDs cover a large group of diseases of heterogeneous etiology, affecting the muscles and/or the nerves and comprising (1) motor neuron disorders [e.g., amyotrophic lateral sclerosis (ALS), spinal muscular atrophy (SMA), and hereditary spastic paraplegia (HSP)], (2) disorders with a component in the peripheral nerves [e.g., Charcot–Marie–Tooth disease (CMT) and giant axonal neuropathy (GAN)], (3) disorders directly affecting the NMJ [e.g., congenital myasthenic syndrome, (CMS) and Lambert–Eaton syndrome], and (4) primary disorders of the muscle (myopathies and muscular dystrophies) (Figure 1B).

While we present numerous zebrafish models generated to model NMDs (Table 1), we do not intend to describe them all. Instead, we focus on highlighting some features as evidence for great relevance and advantage of the zebrafish research model in these types of studies:

**TABLE 1:** Zebrafish models of NMDs and their distinct behavioral and cellular phenotypes.

	Disease	Gene		Zebrafish model	Locomotor phenotype	Cellular phenotype	References
		
		Human	ZF				
**Motor neuron**	**Hereditary spastic paraplegia (HSP):** Upper MN	*SPG3A*	*spg3a*	*spg3a* MO	Impaired touch-evoked escape response (72 hpf)	Abnormal axonal branching and outgrowth	Fassier et al., 2010
		*SPG4*	*spg4*	*spg4* MO	Impaired locomotion and endurance (5 dpf)	Abnormal axonal outgrowth/ NMJ defects (reduction in SV2 and BTX colocalization)	Wood et al., 2006
		*SPG8*	*spg8*	*spg8* MO	N/D	Possible defects in axonal outgrowth	Valdmanis et al., 2007
		*SPG11* *SPG15*	*spg11* *spg15*	*spg11* MO *spg15* MO	N/D	Abnormal axonal branching	Martin et al., 2012
		*SPG39/* *PNPLA6*	*spg39*	*pnpla6* MO	N/D	MN loss/Abnormal axonal branching and outgrowth	Song et al., 2013
		*SPG42/* *SLC33A1*	*slc33a1*	*slc33a1*MO and mutant mRNA	N/D	Abnormal axonal branching and outgrowth	Lin et al., 2008; Mao et al., 2015
		*SPG46/GBA2*	*gba2*	*gba2* MO	Impaired touch-evoked escape response (48 hpf)	Possible defects in axonal outgrowth and branching	Martin et al., 2013
		*SPG53/* *VPS37A*	*vsp37a*	*vsp37a* MO	Impaired touch-evoked escape response (96 hpf)	N/D	Zivony-Elboum et al., 2012
		*SPG76/* *CAPN1*	*capn1a*	*capn1a* MO	N/D	N/D	Gan-Or et al., 2016
		*SPG80/* *UBAP1*	*ubap1*	*ubap1*^–/–^ and mutant RNA	N/D	Possible defects in axonal outgrowth	Farazi Fard et al., 2019
		*IAHSP/* *ALS2*	*als2*	*als2* MO	Impaired touch-evoked escape response (48 hpf)	Possible defects in axonal outgrowth	Gros-Louis et al., 2008
	**Amyotrophic lateral sclerosis (ALS):** Upper and lower MN	*SOD1*	*sod1*	*sod1* mutant mRNA	N/D	Abnormal branching and outgrowth	Lemmens et al., 2007
				*sod1* mutant mRNA	Impaired locomotion upon light stimuli (48 hpf)	Abnormal axonal branching and outgrowth	Robinson et al., 2019
				*sod1^G^* ^93*R*^	Decreased endurance and partial paralysis (10 months)	MN loss/NMJ defects (reduction in SV2 and BTX colocalization)/muscle defects (caliber and degeneration)	Ramesh et al., 2010; Benedetti et al., 2016
				*sod1* ^*T*70I^	Decreased endurance (20 months)	MN loss/NMJ defects (reduction in SV2 and BTX colocalization)	Da Costa et al., 2014
				*sod1^G^* ^93*A*^	Impaired locomotion and endurance (5 months)	MN loss/Abnormal axonal branching and outgrowth/impaired NMJs	Sakowski et al., 2012
		*TDP-43/* *TARDBP*	*tdp-43*	*tdp-43* MO and mutant mRNA	Impaired touch-evoked escape response (48 hpf)	Abnormal axonal branching and outgrowth	Kabashi et al., 2010
				*tdp-43* mutant mRNA	N/D	Abnormal axonal branching and outgrowth	Laird et al., 2010
				*tardbp^–/–^*	N/D	Abnormal axonal outgrowth/disorganization of myofibrils	Schmid et al., 2013
				*tardbp^fh301^*	Impaired escape response (5 dpf)	Abnormal axonal outgrowth	Hewamadduma et al., 2013
		*FUS*	*fus*	*fus* MO and mutant mRNA	Impaired touch-evoked escape response (48 hpf)	Abnormal axonal branching and outgrowth	Kabashi et al., 2011
				*fus* MO and mutant mRNA	Impaired touch-evoked escape response (48hpf)	Reduced CaP rheobase current/NMJ defects (reduction in SV2 and BTX colocalization)	Armstrong and Drapeau, 2013
		*C9ORF72*	*c9orf72*	*c9orf72* MO	Impaired touch-evoked escape response (48 hpf) and spontaneous locomotion (96 hpf)	Abnormal axonal branching and outgrowth	Ciura et al., 2013
				*c9orf72* mutant mRNA	N/D	Abnormal axonal branching and outgrowth	Swinnen et al., 2018
				*c9orf72* ^89*HRE*^	Center avoidance behavior (5 dpf) and decreased endurance (8 months)	MN loss/Muscle atrophy	Shaw et al., 2018
				*c9orf72* ^100*GR*^	Impaired locomotion (7 dpf)	Abnormal axonal branching and outgrowth/Increase apoptosis in the spinal cord	Swaminathan et al., 2018
				*c9orf72* MO	Impaired touch-evoked escape response (48 hpf)	Abnormal axonal branching and outgrowth	Yeh et al., 2018
				*c9orf72^miR^*	Impaired locomotion (6 dpf, 12 months)	MN loss/Reduced AChR clusters at NMJ/Muscle atrophy and defects (weak mEPCs, thin diameter)	Butti et al., 2021
		*SQSTM1*	*sqstm1*	*sqstm1* MO	Impaired touch-evoked escape response(48 hpf)	Abnormal axonal branching and outgrowth	Lattante et al., 2015
	**Spinal muscular atrophy (SMA):** Lower MN	*SMN1,2*	*smn1*	*smn1* MO	N/D	Abnormal axonal outgrowth	McWhorter et al., 2003; Winkler et al., 2005; Gassman et al., 2013; See et al., 2014
				*smn^G^*264*^D^* smn*^Y^*262*^X^* smn*^L^*265*^X^*	N/D	Lack of pre- and post-synaptic protein co-localization/decrease in SV2 protein at the NMJ	Boon et al., 2009
			*smn2*	*smn^Y262–/–^*	Impaired locomotion (9 dpf)	Abnormal axonal branching and outgrowth	Hao et al., 2011, 2012
		*CHODL*	*chodl*	*chodl* MO	N/D	Abnormal axonal branching and outgrowth/Reduced myotome innervation	Zhong et al., 2012
				*chodl* ^–/–^	Impaired touch-evoked escape response (3 dpf)	Prolonged stalling of the CaP axons at the HM/Impaired synaptogenesis	Oprişoreanu et al., 2019, 2021
**Peripheral nerve**	**Charcot-Marie-Tooth disease (CMT2A)**	*MFN2*	*mfn2*	*mfn2* MO	Impaired touch-evoked escape response (48 hpf)	Abnormal axonal outgrowth/U-shaped somites and decreased muscle width	Vettori et al., 2011
				*mfn2* ^L285X/L285X^	Impaired locomotion and endurance (3 months)	NMJ defects (reduction in pre- and post-synaptic area)	Chapman et al., 2013
		*LRSAM1*	*lrsam1*	*lrsam1* MO	Impaired touch-evoked escape response (48 hpf)	N/D	Weterman et al., 2012
		*DNM2*	*dnm2*	*dnm2* MO and mutant mRNA	Impaired touch-evoked escape response (72 hpf)	MN loss/Abnormal axonal branching/NMJ defects (AchR clusters)/increases muscle fiber diameter	Bragato et al., 2016
				*dnm2* ^G537C^	Impaired touch-evoked escape response (48 hpf)	Muscle defects (structural, diameter)	Zhao et al., 2019
	**Giant axonal neuropathy (GAN)**	*GAN*	*gan*	*gan* MO and *gan*^–/–^	Impaired touch-evoked escape response (72 hpf) and spontaneous locomotion (5 dpf)	MN loss (specification defect)/Abnormal axonal branching and outgrowth/NMJ defects/Muscle deficits (U-shape somites, structure)	Arribat et al., 2019
**Neuromuscular junction**	**Congenital myasthenic syndrome (CMS)**	*CHAT*	*chata*	*chata* ^tk64^	Impaired touch-evoked escape response (48 hpf)	No phenotype	Joshi et al., 2018
		*DOK7*	*dok7*	*dok7* MO	Impaired touch-evoked escape response (48 hpf) and spontaneous locomotion (5 dpf)	Abnormal axonal outgrowth/NMJ defects (abnormal AChR prepatterning, reduction in size)/disorganization of slow muscle fiber	Müller et al., 2010; McMacken et al., 2018
		*SLC25A1*	*slc25a1*	*slc25a1* MO	Impaired touch-evoked escape response (48 hpf)	Abnormal axonal outgrowth	Chaouch et al., 2014
		*GFPT1*	*gfpt1*	*gfpt1* MO	Impaired touch-evoked escape response (48 hpf)	Abnormal axonal banching/NMJ defect (delayed development)/Muscle deficits (U-shape somites, structure)	Senderek et al., 2011
		*MYO9A*	*myo9a*	*myo9a* MO	Impaired touch-evoked escape response (48 hpf)	Possible abnormal axonal branching and outgrowth/impaired AChR clustering	O’Connor et al., 2016, 2018
**Muscle**	**Duchenne muscular dystrophy (DMD)**	*DMD*	*dmd*	*dmd* MO	N/D	Disorganized sarcomeres	Guyon et al., 2003
				*sapje*	N/D	Muscle defects (lesions, fiber detachment and retraction, structure, decreased active force)	Bassett et al., 2003; Bassett and Currie, 2004; Widrick et al., 2016
				*dmd^pc2^*	N/D	Muscle defects (fiber detachment and retraction, degeneration)	Berger et al., 2011; Giacomotto et al., 2013
				*sap* ^cl100^	N/D	Muscle degeneration	Guyon et al., 2009
	**Congenital muscular dystrophy**	*ITGA7*	*itg*α*7*	*itg*α*7* MO	N/D	Muscle defect (detachment and retraction of muscle fibers, U-shape somite)	Postel et al., 2008
		*LAMA2*	*lama2*	*caf lama2* ^*cl501/cl501*^	Impaired touch-evoked escape response (72 hpf)	Muscle defects (fiber detachment and retraction, degeneration)	Hall et al., 2007; Gupta et al., 2012
	**Bethlem myopathy**	*COL6A1/COL6A3*	*col6a1/col6a3*	*col6a1* MO *col6a3* MO	Impaired touch-evoked escape response (48 hpf)	Muscle defects (U-shaped somites, structure, degeneration)	Telfer et al., 2010; Zulian et al., 2014; Radev et al., 2015
	**Limb-girdle muscular dystrophy (LGMD)** and **Spinal and bulbar muscular atrophy (SBMA)**	*DNAJB6*	*dnajb6*	*dnajb6* MO and mutant RNA	N/D	Muscle defects (fiber detachment, structure)	Sarparanta et al., 2012; Xu et al., 2022
		*DAG1*	*dag1*	*dag1* MO	Impaired touch-evoked escape response (48 hpf)	Muscle defects (U-shaped somites, fiber detachment, structure, degeneration)	Parsons et al., 2002; Lin et al., 2011; Goody et al., 2012
				*dag1* ^V567D/V567D^	Impaired spontaneous locomotion (7 dpf)	Muscle defects (structure, degeneration)	Gupta et al., 2011
		*POPDC3*	*popdc3*	*popdc3* MO	N/D	Muscle defect (fiber detachment)	Vissing et al., 2019
		*TCAP*	*tcap*	*tcap* MO	Impaired spontaneous locomotion (5 dpf)	Muscle defects (U-shaped somites, fiber detachment, structure)	Zhang et al., 2009; Lv et al., 2022
		*SGCD*	*sgcd*	*sgcd* MO	N/D	Muscle defects (U-shaped somites, structure, degeneration)	Guyon et al., 2005; Cheng et al., 2006
	**LGMD** and **Miyoshi myopathy (MM)**	*DYSF*	*dysf*	*dysf* MO	N/D	Muscle defects (less-clear V-shape somite, structure, degeneration)	Kawahara et al., 2011
	**Dystroglycano-pathies**	*FKRP*	*fkrp*	*fkrp* MO	N/D	Muscle defects (U-shaped somites, fiber detachment, structure, degeneration)	Thornhill et al., 2008; Kawahara et al., 2010; Lin et al., 2011; Bailey et al., 2019
				*fkrp*^Δ^ ^13/Δ^ ^13^	Impaired spontaneous locomotion (5 dpf)	Muscle defect (degeneration)	Serafini et al., 2018
		*FKTN*	*fktn*	*fktn* MO	N/D	Muscle defects (U-shaped somites, degeneration)	Lin et al., 2011
		*INPP5K*	*inpp5k*	*inpp5k* MO	Impaired touch-evoked escape response (72 hpf)	NMJ defect (reduced arborization)/Muscle defects (structure, degeneration)	Osborn et al., 2017
		*ISPD*	*ispd*	*ispd* MO	N/D	Muscle defects (retracting fibers, structure, degeneration)	Roscioli et al., 2012
		*B3GNT1*	*b3gnt*	*b3gnt1* MO	N/D	Muscle defects (fiber detachment, U-shaped somites, structure, degeneration)	Buysse et al., 2013
		*GTDC2*	*gtdc2*	*gtdc2* MO	N/A	Possible muscle defect (U-shaped somites)	Manzini et al., 2012
	**Myotubular myopathy**	*MTM1*	*mtm1*	*mtm1* MO	Impaired touch-evoked escape response (72 hpf)	Muscle defects (fiber detachment, structure, excitation-contraction coupling abnormalities)	Dowling et al., 2009
	**Centronuclear myopathies (CNMs)**	*MTMR14*	*mtmr14*	*mtmr14* MO	Impaired touch-evoked escape response (48 hpf)	Muscle defect (excitation-contraction coupling abnormalities)	Dowling et al., 2010
		*DNM2*	*dnm2*	*dnm2* mutant RNA	Impaired touch-evoked escape response (72 hpf)	NMJ defects (defects in AChR clustering)/Muscle defects (structure)	Gibbs et al., 2013
	**Myofibrillar myopathy**	*DESM*	*desm*	*desma* MO *desmb* MO	Impaired spontaneous locomotion (4 dpf)	Muscle defects (fiber detachment, structure, decreased active force)	Li et al., 2013
		*FLNC*	*flnc*	*sot* Mutant *flnca* MO *flnc^W^*^2710*X*^	N/D	Muscle defects (fiber disintegration, structure, degeneration)	Ruparelia et al., 2012, 2016
		*ZASP*	*zasp*	*cypher* MO	N/D	Muscle defects (U-shaped somites, structure, degeneration)	van der Meer et al., 2006
	**Congenital myopathy**	*RYR1*	*ryr1b*	*ryr^mi^* ^340^	Impaired touch-evoked escape response (48 hpf)	Muscle defects (structure, excitation-contraction coupling abnormalities)	Hirata et al., 2007

Here we focus on neuromuscular phenotypes and excluded other phenotypes (cardiac, central nervous system alterations…) described in the articles. We also exclude specific form of HSP (MARS, X-linked formed): see Naef et al. (2019) for complete review. ZF, zebrafish; MO, morpholino; N/D, not determined (or data not provided); HM, horizontal myoseptum; mEPCs, miniature endplate currents; BTX, bungarotoxin.

- Zebrafish models of the same disease group, for example, of the MNs (ALS, SMA, and HSP) induce similar deficits, including loss of MNs, aberrant axonal outgrowth, loss of neuromuscular connectivity, muscle denervation, and defective motor performance. The study of these parameters, as easily performed in the zebrafish, enriches our knowledge of the molecular players in the settings and maintenance of the neuromuscular system.
- In a context of diseases with multiple genetic origins (most of the clinical groups described in Table 1), the zebrafish model provides the versatility to validate gene discovery and pathogenic variants using fast and penetrant functional characterization (see Patten et al., 2014; Naef et al., 2019 for review).
- For several diseases, such as GAN (Arribat et al., 2019) and HSP (als2, Gros-Louis et al., 2008), zebrafish models are the first to reproduce the severity of motor and neuromuscular symptoms, where mouse models have so far failed (Cai et al., 2008; Dequen et al., 2008; Ganay et al., 2011). This reinforces the notion of the conservation of the neuromuscular system between the zebrafish and human species.
- The zebrafish represents a highly relevant model for therapeutic development as its physiology is well conserved with human. For example, the zebrafish is an excellent model for muscle pathologies (Jagla et al., 2017), more adapted than mouse (Berger and Currie, 2012). Models with mutations in the zebrafish dystrophin gene (*dmd* and *sapje* models) of DMD have been developed (Bassett and Currie, 2004; Guyon et al., 2009), whose robustness of phenotypes (muscle lesions and birefringence, almost 100% penetrant) has led to the development of a therapeutic strategy for human patients (Johnson et al., 2013; Farr et al., 2020), which is now in phase 3 in clinical trial.

## Current methods to study the neuromuscular system in health and in neuromuscular diseases

The phenotyping of zebrafish models of NMDs and, more generally, the study on the regulation(s) of the neuromuscular system rely on two levels of analysis: the locomotion at the behavioral level (Granato et al., 1996) and the visualization of the NMJ at the cellular level (Fetcho, 2007). We present the most common tools used in zebrafish, highlighting their advantages and their limitations (Figure 1C).

### Locomotion assay: First level of investigation

The zebrafish larvae serve as a powerful model to dissect locomotor patterns as their swimming behavior is defined by sequences of stereotyped movements (Wolman and Granato, 2012). At 5 dpf, when the neuromuscular system is established, the zebrafish is capable of fully autonomous and spontaneous movement. At this stage, motility assays have the advantage to be purely motor, contrary to other locomotor tests performed on younger zebrafish embryos, which include a sensory component (e.g., evoked response to stimuli at 48 hpf). The analysis of locomotor activity of larval zebrafish has emerged as a potent tool for phenotypic assessment in the neurosciences and toxicology fields (Basnet et al., 2019). Some commercial tools are available to the community (e.g., Noldus, Viewpoint, or Loligo^®^ Systems): they provide a means to measure different properties of the swimming activity, including quantification of kinetic parameters such as frequency, duration, speed, and total distance traveled within a given time (Noldus et al., 2001). Few academic laboratories developed locomotion-based tracking systems with deeper analysis of the movement patterns of larvae that are suitable for high-content analysis (Colwill and Creton, 2011; Zhou et al., 2014). They were instrumental in providing a better analysis of the sequencing of swimming bouts (Reddy et al., 2022) and in discriminating the great variability in the escape behaviors (Kohashi and Oda, 2008), by measuring the delay, amplitude, duration, frequency, angle, and number of the tail-bending oscillations (Mirat et al., 2013). Overall, the latter methodologies are extremely attractive, translationally relevant, and easily adaptable to high-throughput pharmacological screening strategies but unfortunately still too rarely used by the general academic community.

While important for the comprehension of the locomotor network (Berg et al., 2018), behavioral tests are not systematically used in disease models, as shown in Table 1. Still, when performed, they are valuable to quantify the degree of locomotor deficits: less frequent turning and swimming bouts in the SMA zebrafish model (Hao et al., 2012), shorter traveled distance in the ALS zebrafish model (Armstrong and Drapeau, 2013; Robinson et al., 2019), and severe reduction in both total distance and net velocity (Zhao et al., 2019) in CMT and GAN models (Arribat et al., 2019). Nevertheless, these tools present limitations when it comes to grading the severity of dysfunction between NMD types, or for distinct genes within the same group of diseases. This challenge is mainly due to high variability in the settings applied to the motility tests (type, alternation of light/dark stimulation, duration of the assay, etc.). For example, one can note that the great disparity in the locomotor tests used to describe motor deficits in ALS models, specifically in the *SOD1* gene (see Table 1). This aspect has some importance when choice needs to be made to select strong candidates for preclinical studies or discuss the relative contribution of molecular players. Most importantly, current behavioral tests have limited outcomes in defining the functional origin (neuron, NMJ, or muscle) and the cellular signature of individual gene or NMD.

### Cellular defects: 2d level to provide specificity

Due to their transparency, zebrafish embryos are particularly suited for cellular imaging (see Pappalardo et al., 2013; Babin et al., 2014; Patten et al., 2014 for review). For the motor system, classic methods are the labeling of the sub-compartments of the NMJ (Panzer et al., 2005) and providing qualitative measures of the integrity and stability of the NMJ. Some pieces of off-line, free, and open-source image processing and analysis software have been used as quantitative tools for the analysis of the NMJ such as axonal length, AChR clustering, and the coefficient of co-localization of NMJ markers (pre- and postsynaptic). Thus, from confocal images, the authors use these methods to (1) measure axonal length [e.g., NeuronJ plugin in ImageJ software (Boon et al., 2009; Robinson et al., 2019; Campanari et al., 2021; Oprişoreanu et al., 2021) or Lucia software (Lemmens et al., 2007)] or (2) define the score for normal versus abnormal (McWhorter et al., 2003; Boyd et al., 2017; Swinnen et al., 2018). Rare laboratories have developed specific metrics for the quantification of motor axon development, as in Smn-deficient zebrafish embryos (Gassman et al., 2013). Overall, the existing analysis tools permit the extraction of quantifiable data of only a limited number (about six or seven) of axons per zebrafish sample. Thus, one important limitation of the current cellular analysis is the difficulty in extracting measurable data for the whole NMJ, especially due to the overlap between pre- and postsynaptic staining. Indeed, unlike other mammalian species such as the mouse, the fish exhibits a spreaded distribution of the motor nerve terminals and a great extent of postsynaptic folding (Slater, 2017), thus particularly challenging to visualize in detail and quantify.

## Perspective in cellular imaging

Currently, most cellular studies in zebrafish are conducted manually, using low-throughput imaging techniques. Still, there is a need for standardized, quantitative, and high-resolution techniques to gain in-depth knowledge of the neuromuscular unit and to define specificity of alterations of NMJ (and therefore mechanisms) across pathologies. Moreover, similar to behavioral assays, developing automated tools, compatible with high-throughput screening assays, would be essential to identify and determine the effectiveness of potential drugs in modulating or restoring the neuromuscular system in health and disease. Recent advancements in imaging-based high-content screening technologies for drug discovery and toxicology have mostly found applications in 3D tissue culture or 3D organoid models (Li et al., 2016). Although the zebrafish has earned its place as an efficient model for high-throughput drug screening, the current tools lack a standard pipeline for imaging small organisms. Therefore, designing a quantifiable and standardized methodology with robust assay metrics to quantify parameters of the neuromuscular unit would be critical for both fundamental biology and clinical research.

## Development of a novel quantitative imaging methodology to study and treat the neuromuscular system

To address the challenge of standardization and reproducibility of imaging tools in zebrafish, we developed an automated, high-throughput, imaging pipeline in zebrafish, integrating a quantitative analysis of the key parameters of the neuromuscular system: axonal length, AChR clustering, and NMJ synapse. Here, we mainly describe this novel quantitative methodology and briefly illustrate its potency in delineating the specific phenotype and identifying therapeutic drugs in GAN disease.

**FIGURE 2:**
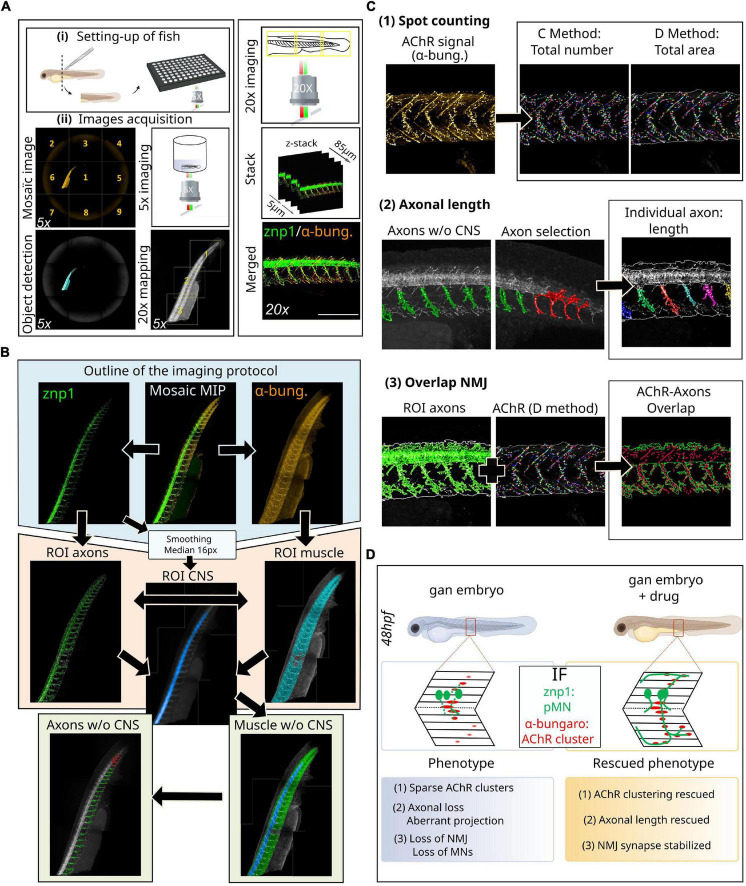
Novel methodology for high-throughput imaging of the neuromuscular system in the zebrafish. **(A)** Schematic overview of the setup of 48hpf larvae in 96-well plates (i) for automated detection and image acquisition (ii), from object detection (5x) to stacked images (20x). **(B)** Outline of the imaging and analytic protocol: representative images of NMJ staining (znp1 in green, α-bungarotoxin in red) within the spinal cord of control larvae, from which several steps of ROI segmentation allow to define the different components of the NMJ. **(C)** Representative images of the different filters resulting in the segmentation of AChR clusters from α-bungarotoxin staining and individual axon from the znp1 staining, enabling the quantitative assessment of spot counting (1), axonal length (2), and NMJ overlapping compounds within the spinal cord (3). See the Supplementary Figure 1 to assess for the quality of the processing tools for each filter: raw data compared to post-analysis pictures at high magnification. **(D)** Validation of this methodology in the zebrafish model of giant axonal neuropathy (*gan*). Detailed coverage of NMJ defects in the gan model and identification of hits restoring three parameters of the neuromuscular system, following a high-throughput screening. α-bung, α-bungarotoxin; pMN, primary motor neuron; MN, motor neuron; NMJ, neuromuscular junction; AChR, acetylcholine receptors; MIP, maximum intensity projections; ROI, region of interest; w/o, without; CNS, central nervous system.

Our method relies on automated high-resolution image acquisition using multi-well plates (Opera Phenix™ high-content imaging systems, Perkin Elmer) and involves the development of an image processing sequence using Harmony software (v4.9, Perkin Elmer) to prefilter whole-embryo images and exclude regions with artifacts and high background. This protocol allows to define the regions of interest (ROIs) within the spinal cord and make them automatically detectable and accessible to downstream analysis (Figure 2).

• Prerequisite: The first prerequisite concerns the establishment of a model of the neuromuscular system or pathology in the zebrafish, whose phenotype is at least partially described in larval stages. It can include a motor component, with a defect in locomotion with a severity ideally similar to that observed in human patients and/or a cellular defect of varying severity in the neuromuscular system.
• Possible applications: The methodology presented here can have a fundamental and/or therapeutic vocation. Indeed, it allows to scrutinize the neuromuscular phenotypes in greater detail and to identify therapeutic molecules able to restore them. In the latter, it is recommended to carefully select a chemical library of interest (see Peterson and Fishman, 2011 for review).
• Procedure/Methodology: In the next paragraph, we provide a detailed description of the high-throughput imaging approach, which offers high-resolution images of 3D zebrafish larvae mounted in individual wells in 96-well plates. Then, we detail the development of an image analysis sequence, allowing first to define the different ROIs by using maximum intensity projections (MIP) and then to submit them to different analysis filters to extract measurable neuromuscular parameters. Noteworthy, our methodology on high-resolution images allows the quantification of the NMJ, a challenge in the zebrafish model due to dispersed sites of muscle innervation.

### Setting up of fish

Zebrafish embryos of desired aged (see “Advantages of the method” section) are placed in 96-well plates and eventually exposed to small-molecule libraries of compounds that can elicit phenotypic alterations (Figure 2Ai). While three larvae represent the most common settings (Rennekamp and Peterson, 2015), we determined that each well can house up to four zebrafish larvae without compromising proper development. On another note, it is recommended to use plates with a thin and flat plastic or glass bottom. Since the interest here is to image a lateral view of the spinal cord, we recommend cutting the embryo at the anterior part of the yolk extension to enable lateral positioning.

### Axonal and neuromuscular junction staining

Neuromuscular junctions can be visualized by light microscopy following labeling of both the presynaptic nerve terminal and the postsynaptic acetylcholine receptors (Figure 2A). The most commonly used presynaptic antigens, as in other vertebrate models, include anti-neurofilament (e.g., 3A10), anti-synaptotagmin (e.g., znp-1), anti-neurolin (e.g., zn8), and anti-synaptic vesicle glycoprotein 2 (SV2) to fully label both the pre-synaptic axon and nerve terminal. The fluorescently tagged α-bungarotoxin is the most commonly used protein to label acetylcholine receptors in the postsynaptic membranes of the NMJ. Here, we opted to use the znp-1/α-bungarotoxin combination, which ensures specific marking of the axons and NMJ with little background. Primary antibodies are from the following sources: mouse IgG2a anti-synaptotagmin (1:100, znp-1, DSHB) and anti-α-bungarotoxin (1:50, B35451, Invitrogen). We believe the same methodology can be applied to other NMJ markers mentioned earlier. Indeed, the analysis sequences permit the collection of a large number of measurable parameters, as long as the immunostaining is of required quality.

### Image acquisition settings of chemical-treated embryos

1. Pre-scan is performed at 5X magnification on the full well surface (nine fields per well with 6% overlap) and red channel only (561 nm) with the widefield mode (Figure 2Aii).
2. Use the “*In-the-fly image analysis*” tool to create a mosaic image and “*Find Image Region*” module to detect the whole zebrafish.
3. Use the “*Determine Well Layout*” module to define a 20X re-scan magnification (with 6% overlap) covering the entire object.
4. Re-scan at 20X magnification on whole fish larvae in the confocal mode. Green (488 nm/znp-1) and red (561 nm/α-bungarotoxin) channels are imaged, with a z-stack of 85 μm (5 μm interval).

### Definition of regions of interest

5. First, acquire mosaic images (full fish larvae) with MIP for each channel (Figure 2B).
6. To automatically identify the two ROI named axons and muscle, we used the “*Find Image Region*” module in green (488 nm/znp-1) and red (561 nm/α-bungarotoxin) channels, respectively. The final ROI muscle was obtained by subtracting seven pixels around the initial region to restrict the analysis to the region of interest and to reduce detection of artifacts.
7. The main challenge is to separate each axon into a different entity. To do this, it is imperative to differentiate the axons from the spinal cord. From the same mosaic images with MIP, we applied smoothing with a median filter of 20 px using the “*Filter Image*” module in the green (488 nm/znp-1) channel. On this new image, we used the “*Find Image Region*” module to find a new ROI: central nervous system (CNS, spinal cord). Then, we used “*Calculate Morphology Properties*” to measure the size and the position of the CNS ROI.
8. Using the “*Find Surrounding Region*” module, the CNS ROI was subtracted from the fish body area (ROI muscle), to create a new surrounding region: body without CNS.

### Image analysis settings

#### Acetylcholine receptors quantification

9. Acetylcholine receptors localization/quantification was performed in the ROI muscle using the “*Find Spots*” module on the α-bungarotoxin channel [Figure 2C(1)]. A total of two distinct methods were used to extract the properties of the AChR spots: method C, more sensitive to the detection of the exact number, and method D, more sensitive to the detection of the area. It is also possible to extract raw data on the number of AChR clusters (method C) and their area (method D) in px^2^ or μm^2^, as well as their average labeling intensity (see Supplementary Figure 1).

#### Axonal length

10. Using the “*Find Image Region*” module only in the surrounding region (body without the CNS), we created a new ROI: axons without the CNS, corresponding to the axonal region without the spinal cord [Figure 2C(2)]. This is particularly important because the bright spinal cord signal hampers the detection of weaker axonal staining. With this restricted region, we applied a “*Modify Population*” module with “*Cluster by Distance*” (2 px distance and area > 300 px^2^) to individualize axons. An axon selection step can be added to eliminate detection problems with the “*Selection Population*” module (in case some axons have not been properly individualized).

11. Finally, we used the “*Calculate Morphology Properties*” module to measure individual axonal length. The data are presented as length units (μm) for length and width and μm^2^ for axonal area (see Supplementary Figure 1).

#### Overlap neuromuscular junction

12. To measure AChR/axon overlap, we used the “*Calculate Position Properties - Cross Population*” module to obtain the overlap percentage between the AChR total area and axon ROI [Figure 2C(3)]. This calculation corresponds to the percentage of the AChR cluster area included in the axon ROI area compared to the total AChR area. It is built on the raw data of the axon ROI area (μm^2^) and the postsynaptic AChR area (μm^2^) calculated using the method D (see Supplementary Figure 1). To enable the reproducibility of data and use of the analysis sequences obtained by the Harmony software (Perkin Elmer), all the pre-prints are added in HTML format as Supplementary Datasheet 1.

• Validation of this methodology (Figure 2D): We validated this novel methodology with the zebrafish model of giant axonal neuropathy. GAN is a fatal disease, presenting a widespread phenotype which starts with a peripheral neuropathy in infancy and extends to the brain in young adults (Lescouzères and Bomont, 2020) (Figure 1B and Table 1). Since we identified gigaxonin as the defective protein in this disease (Bomont et al., 2000), we have generated a robust zebrafish model of the pathology by (1) transiently repressing gigaxonin expression using morpholino antisense oligonucleotides and (2) creating a knockout line. Both zebrafish models reproduce the loss of motility described in GAN patients (Arribat et al., 2019). Using our high-resolution and quantitative imaging methodology, we refined the role of gigaxonin in controlling (1) AChR clustering, (2) axonal outgrowth/projection, and (3) stability of the NMJ. In addition, the combination of this novel high-content image analysis pipeline with high-throughput screening allowed us to pinpoint drugs capable of restoring all three parameters and develop a therapeutic scheme for preclinical studies (Lescouzères et al., Submitted).
• Advantages of the method: One of the main advantages of this approach is the generation of standardized and quantifiable data for parameters that are usually assessed with either qualitative measures or manual and tedious quantitative tools. In particular, the intention is to address the challenge of quantitative analysis of the NMJ with high throughput. Here, the analysis is automated and allows for robust characterization of a novel phenotype and/or the testing of a large number of compounds in drug screening. Moreover, since the data are obtained from a large number of larvae, it is possible to collect more robust statistics in different populations (diseased/treated larvae, between different genes, etc.). For diseased embryos with modest or less penetrant phenotype, the statistical power of such analysis is extremely high. In addition, our methodology enables the establishment of thresholds from which a specific NMJ-associated parameter is considered affected and/or restored in the treated diseased embryos. Thus, this novel imaging methodology designed for the neuromuscular system presents various advantages that only require a precise positioning of the zebrafish embryos in wells. Noteworthy, the imaging methodology we propose has no time limit and may, in principle, allow the observation of early events of the neuromuscular contacts. To study MN differentiation, (1) the first wave of primary motor neurons at the end of gastrulation (9–10 hpf) or (2) the second wave of secondary motor neurons (14–15 hpf) can be imaged using islet staining (Eisen et al., 1986; Westerfield et al., 1986). Prior to the establishment of motor nerve terminal contacts, postsynaptic muscle fibers form primitive, dynamic, and non-synaptic AChR clusters that are distributed on the membrane of the adaxial cells. This step, called muscle pre-patterning (Panzer et al., 2005; Wu et al., 2010), can be specifically investigated with the α-bungarotoxin staining between 14 and 16 hpf. In addition, axonal branching and outgrowth are studied at more advanced stages, that is, 26–30 hpf for the pMNs (CaP, MiP, and RoP) and 30–50 hpf for sMNs. (Myers, 1985) using znp1 and zn8 staining, respectively. Finally, this workflow for high-throughput image analysis could be easily adapted to study other tissues, such as the retina in the zebrafish. This adaptation would only require the selection of sufficiently specific antibodies, to ensure the reproducible detection thresholds from one embryo to another.

## Conclusion and future prospects

The zebrafish species shares great genetic similarities with humans and is a model of choice for studying the physiology of the neuromuscular system. On the one hand, in the fundamental field, the access to all embryological stages and the ease of use of the model (imaging and housing) allow for the integration of the zebrafish in many comparative studies and the identification of key molecular players in the neuromuscular system. In the field of human genetics, the zebrafish has proven its strength for diagnosis purpose, gene identification, and modeling diseases of the motor neuron, NMJ, and muscle. Taking advantage of the small size and optic transparency of the embryo/larva, behavioral and cellular methodologies uncovered fundamental and disease mechanisms sustaining motor functions. Still, limitations lie in the strength of the assays as one could argue that all NMDs or molecular players look similar, with altered locomotion and decreased axonal outgrowth. Increasing precision is not only necessary to fully decipher the molecular pathways controlling the neuromuscular unit but also mandatory to differentiate diseases and offer the specificity required for personalized medicine. While behavioral assays have been enriched to quantify various parameters of motility in an automated manner, current imaging protocols do not reach this level of development. Here, we present a novel, standardized, automated, and quantitative imaging pipeline for the key parameters of the neuromuscular unit, including the so far challenging quantification of the NMJ. As validated in our GAN disease model, we expect our imaging and analysis tools to be broadly useful to researchers interested in scrutinizing the motor system in detail and executing high-throughput screening in zebrafish models of NMDs.

## Data availability statement

The original contributions presented in this study are included in the article/Supplementary material, further inquiries can be directed to the corresponding author.

## Ethics statement

This animal study was reviewed and approved by the French ministry (reference N°036) for the creation of the gan zebrafish line.

## Author contributions

PB contributed to the conception and design of the work, drafting the manuscript and figures, and critical revisions of the manuscript. LL and BB generated and designed the figures. LL executed the drawing of figures. All authors performed the bibliography search and wrote and reviewed the manuscript.

## References

[B1] Armstrong G. A. B. Drapeau P. (2013). Loss and gain of FUS function impair neuromuscular synaptic transmission in a genetic model of ALS. *Hum. Mol. Genet.* 22 4282–4292. 10.1093/hmg/ddt278 23771027

[B2] Arribat Y. Mysiak K. S. Lescouzères L. Boizot A. Ruiz M. Rossel M. (2019). Sonic hedgehog repression underlies gigaxonin mutation-induced motor deficits in giant axonal neuropathy. *J. Clin. Invest.* 129 5312–5326. 10.1172/JCI129788 31503551 PMC6877328

[B3] Babin P. J. Goizet C. Raldúa D. (2014). Zebrafish models of human motor neuron diseases: Advantages and limitations. *Progr. Neurobiol.* 118 36–58. 10.1016/j.pneurobio.2014.03.001 24705136

[B4] Bailey E. C. Alrowaished S. S. Kilroy E. A. Crooks E. S. Drinkert D. M. Karunasiri C. M. (2019). NAD+ improves neuromuscular development in a zebrafish model of FKRP-associated dystroglycanopathy. *Skelet. Muscle* 9:21. 10.1186/s13395-019-0206-1 31391079 PMC6685180

[B5] Banerjee S. Gordon L. Donn T. M. Berti C. Moens C. B. Burden S. J. (2011). A novel role for MuSK and non-canonical Wnt signaling during segmental neural crest cell migration. *Development* 138 3287–3296. 10.1242/dev.067306 21750038 PMC3133918

[B6] Barbazuk W. B. (2000). The syntenic relationship of the zebrafish and human genomes. *Genome Res.* 10 1351–1358. 10.1101/gr.144700 10984453 PMC310919

[B7] Basnet R. M. Zizioli D. Taweedet S. Finazzi D. Memo M. (2019). Zebrafish larvae as a behavioral model in neuropharmacology. *Biomedicines* 7:E23. 10.3390/biomedicines7010023 30917585 PMC6465999

[B8] Bassett D. I. Bryson-Richardson R. J. Daggett D. F. Gautier P. Keenan D. G. Currie P. D. (2003). Dystrophin is required for the formation of stable muscle attachments in the zebrafish embryo. *Development* 130 5851–5860. 10.1242/dev.00799 14573513

[B9] Bassett D. Currie P. D. (2004). Identification of a zebrafish model of muscular dystrophy. *Clin. Exp. Pharmacol. Physiol.* 31 537–540. 10.1111/j.1440-1681.2004.04030.x 15298547

[B10] Beattie C. E. (2000). Control of motor axon guidance in the zebrafish embryo. *Brain Res. Bull.* 53 489–500. 10.1016/S0361-9230(00)00382-8 11165784

[B11] Beattie C. E. Hatta K. Halpern M. E. Liu H. Eisen J. S. Kimmel C. B. (1997). Temporal separation in the specification of primary and secondary motoneurons in zebrafish. *Dev. Biol.* 187 171–182. 10.1006/dbio.1997.8604 9242415

[B12] Bello-Rojas S. Istrate A. E. Kishore S. McLean D. L. (2019). Central and peripheral innervation patterns of defined axial motor units in larval zebrafish. *J. Comp. Neurol.* 527 2557–2572. 10.1002/cne.24689 30919953 PMC6688944

[B13] Benedetti L. Ghilardi A. Rottoli E. De Maglie M. Prosperi L. Perego C. (2016). INaP selective inhibition reverts precocious inter- and motorneurons hyperexcitability in the Sod1-G93R zebrafish ALS model. *Sci. Rep.* 6:24515. 10.1038/srep24515 27079797 PMC4832213

[B14] Berg E. M. Björnfors E. R. Pallucchi I. Picton L. D. El Manira A. (2018). Principles governing locomotion in vertebrates: Lessons from zebrafish. *Front. Neural Circuits* 12:73. 10.3389/fncir.2018.00073 30271327 PMC6146226

[B15] Berger J. Currie P. D. (2012). Zebrafish models flex their muscles to shed light on muscular dystrophies. *Dis. Model Mech.* 5 726–732. 10.1242/dmm.010082 23115202 PMC3484855

[B16] Berger J. Berger S. Jacoby A. S. Wilton S. D. Currie P. D. (2011). Evaluation of exon-skipping strategies for Duchenne muscular dystrophy utilizing dystrophin-deficient zebrafish. *J. Cell Mol. Med.* 15 2643–2651. 10.1111/j.1582-4934.2011.01260.x 21251213 PMC4373433

[B17] Boehm I. Alhindi A. Leite A. S. Logie C. Gibbs A. Murray O. (2020). Comparative anatomy of the mammalian neuromuscular junction. *J. Anat.* 237 827–836. 10.1111/joa.13260 32573802 PMC7542190

[B18] Bomont P. Cavalier L. Blondeau F. Hamida C. B. Belal S. Tazir M. (2000). The gene encoding gigaxonin, a new member of the cytoskeletal BTB/kelch repeat family, is mutated in giant axonal neuropathy. *Nat. Genet.* 26 370–374. 10.1038/81701 11062483

[B19] Boon K.-L. Xiao S. McWhorter M. L. Donn T. Wolf-Saxon E. Bohnsack M. T. (2009). Zebrafish survival motor neuron mutants exhibit presynaptic neuromuscular junction defects. *Hum. Mol. Genet.* 18 3615–3625. 10.1093/hmg/ddp310 19592581 PMC2742401

[B20] Boyd P. J. Tu W.-Y. Shorrock H. K. Groen E. J. N. Carter R. N. Powis R. A. (2017). Bioenergetic status modulates motor neuron vulnerability and pathogenesis in a zebrafish model of spinal muscular atrophy. *PLoS Genet.* 13:e1006744. 10.1371/journal.pgen.1006744 28426667 PMC5417717

[B21] Bragato C. Gaudenzi G. Blasevich F. Pavesi G. Maggi L. Giunta M. (2016). Zebrafish as a model to investigate dynamin 2-related diseases. *Sci. Rep.* 6:20466. 10.1038/srep20466 26842864 PMC4740890

[B22] Brooke M. H. Kaiser K. K. (1970). Muscle fiber types: How many and what kind? *Arch. Neurol.* 23 369–379. 10.1001/archneur.1970.00480280083010 4248905

[B23] Burke R. E. Levine D. N. Zajac F. E. (1971). Mammalian motor units: Physiological-histochemical correlation in three types in cat gastrocnemius. *Science* 174 709–712. 10.1126/science.174.4010.709 4107849

[B24] Butti Z. Pan Y. E. Giacomotto J. Patten S. A. (2021). Reduced C9orf72 function leads to defective synaptic vesicle release and neuromuscular dysfunction in zebrafish. *Commun. Biol.* 4:792. 10.1038/s42003-021-02302-y 34172817 PMC8233344

[B25] Buysse K. Riemersma M. Powell G. van Reeuwijk J. Chitayat D. Roscioli T. (2013). Missense mutations in β-1,3-N-acetylglucosaminyltransferase 1 (B3GNT1) cause walker-warburg syndrome. *Hum. Mol. Genet.* 22 1746–1754. 10.1093/hmg/ddt021 23359570 PMC3613162

[B26] Cai H. Shim H. Lai C. Xie C. Lin X. Yang W. J. (2008). ALS2/alsin knockout mice and motor neuron diseases. *Neurodegener. Dis.* 5 359–366. 10.1159/000151295 18714162 PMC2556598

[B27] Campanari M.-L. Marian A. Ciura S. Kabashi E. (2021). TDP-43 regulation of AChE expression can mediate ALS-Like phenotype in zebrafish. *Cells* 10:221. 10.3390/cells10020221 33499374 PMC7911940

[B28] Chaouch A. Porcelli V. Cox D. Edvardson S. Scarcia P. De Grassi A. (2014). Mutations in the mitochondrial citrate carrier SLC25A1 are associated with impaired neuromuscular transmission. *J. Neuromuscul. Dis.* 1 75–90. 10.3233/JND-140021 26870663 PMC4746751

[B29] Chapman A. L. Bennett E. J. Ramesh T. M. De Vos K. J. Grierson A. J. (2013). Axonal transport defects in a mitofusin 2 loss of function model of charcot-marie-tooth disease in zebrafish. *PLoS One* 8:e67276. 10.1371/journal.pone.0067276 23840650 PMC3694133

[B30] Cheng L. Guo X. Yang X. Chong M. Cheng J. Li G. (2006). Delta-sarcoglycan is necessary for early heart and muscle development in zebrafish. *Biochem. Biophys. Res. Commun.* 344 1290–1299. 10.1016/j.bbrc.2006.03.234 16650823

[B31] Ciura S. Lattante S. Le Ber I. Latouche M. Tostivint H. Brice A. (2013). Loss of function of C9orf72 causes motor deficits in a zebrafish model of amyotrophic lateral sclerosis. *Ann. Neurol.* 74 180–187. 10.1002/ana.23946 23720273

[B32] Colwill R. M. Creton R. (2011). Locomotor behaviors in zebrafish (Danio rerio) larvae. *Behav. Process.* 86 222–229. 10.1016/j.beproc.2010.12.003 21147203 PMC3063417

[B33] Da Costa M. M. J. Allen C. E. Higginbottom A. Ramesh T. Shaw P. J. McDermott C. J. (2014). A new zebrafish model produced by TILLING of SOD1-related amyotrophic lateral sclerosis replicates key features of the disease and represents a tool for in vivo therapeutic screening. *Dis. Model Mech.* 7 73–81. 10.1242/dmm.012013 24092880 PMC3882050

[B34] Dequen F. Bomont P. Gowing G. Cleveland D. W. Julien J.-P. (2008). Modest loss of peripheral axons, muscle atrophy and formation of brain inclusions in mice with targeted deletion of gigaxonin exon 1. *J. Neurochem.* 107 253–264. 10.1111/j.1471-4159.2008.05601.x 18680552 PMC3657508

[B35] Devoto S. H. Melançon E. Eisen J. S. Westerfield M. (1996). Identification of separate slow and fast muscle precursor cells in vivo, prior to somite formation. *Development* 122 3371–3380. 10.1242/dev.122.11.3371 8951054

[B36] Dowling J. J. Low S. E. Busta A. S. Feldman E. L. (2010). Zebrafish MTMR14 is required for excitation-contraction coupling, developmental motor function and the regulation of autophagy. *Hum. Mol. Genet.* 19 2668–2681. 10.1093/hmg/ddq153 20400459 PMC2883342

[B37] Dowling J. J. Vreede A. P. Low S. E. Gibbs E. M. Kuwada J. Y. Bonnemann C. G. (2009). Loss of myotubularin function results in T-tubule disorganization in zebrafish and human myotubular myopathy. *PLoS Genet.* 5:e1000372. 10.1371/journal.pgen.1000372 19197364 PMC2631153

[B38] Efthymiou S. Manole A. Houlden H. (2016). Next-generation sequencing in neuromuscular diseases. *Curr. Opin. Neurol.* 29 527–536. 10.1097/WCO.0000000000000374 27588584 PMC5082606

[B39] Eisen J. S. Smith J. C. (2008). Controlling morpholino experiments: Don’t stop making antisense. *Development* 135 1735–1743. 10.1242/dev.001115 18403413

[B40] Eisen J. S. Myers P. Z. Westerfield M. (1986). Pathway selection by growth cones of identified motoneurones in live zebra fish embryos. *Nature* 320 269–271. 10.1038/320269a0 3960108

[B41] Farazi Fard M. A. Rebelo A. P. Buglo E. Nemati H. Dastsooz H. Gehweiler I. (2019). Truncating mutations in UBAP1 cause hereditary spastic paraplegia. *Am. J. Hum. Genet.* 104 767–773. 10.1016/j.ajhg.2019.03.001 30929741 PMC6451742

[B42] Farr G. H. Morris M. Gomez A. Pham T. Kilroy E. Parker E. U. (2020). A novel chemical-combination screen in zebrafish identifies epigenetic small molecule candidates for the treatment of Duchenne muscular dystrophy. *Skelet. Muscle* 10:29. 10.1186/s13395-020-00251-4 33059738 PMC7559456

[B43] Fassier C. Hutt J. A. Scholpp S. Lumsden A. Giros B. Nothias F. (2010). Zebrafish atlastin controls motility and spinal motor axon architecture via inhibition of the BMP pathway. *Nat. Neurosci.* 13 1380–1387. 10.1038/nn.2662 20935645

[B44] Fetcho J. R. (2007). The utility of zebrafish for studies of the comparative biology of motor systems. *J. Exp. Zool. B Mol. Dev. Evol.* 308 550–562. 10.1002/jez.b.21127 17024661

[B45] Gaj T. Gersbach C. A. Barbas C. F. (2013). ZFN, TALEN, and CRISPR/Cas-based methods for genome engineering. *Trends Biotechnol.* 31 397–405. 10.1016/j.tibtech.2013.04.004 23664777 PMC3694601

[B46] Ganay T. Boizot A. Burrer R. Chauvin J. Bomont P. (2011). Sensory-motor deficits and neurofilament disorganization in gigaxonin-null mice. *Mol. Neurodegener.* 6:25. 10.1186/1750-1326-6-25 21486449 PMC3094382

[B47] Gan-Or Z. Bouslam N. Birouk N. Lissouba A. Chambers D. B. Vérièpe J. (2016). Mutations in CAPN1 cause autosomal-recessive hereditary spastic paraplegia. *Am. J. Hum. Genet.* 98 1038–1046. 10.1016/j.ajhg.2016.04.002 27153400 PMC4863665

[B48] Gassman A. Hao L. T. Bhoite L. Bradford C. L. Chien C.-B. Beattie C. E. (2013). Small molecule suppressors of Drosophila kinesin deficiency rescue motor axon development in a zebrafish model of spinal muscular atrophy. *PLoS One* 8:e74325. 10.1371/journal.pone.0074325 24023935 PMC3762770

[B49] Giacomotto J. Brouilly N. Walter L. Mariol M.-C. Berger J. Ségalat L. (2013). Chemical genetics unveils a key role of mitochondrial dynamics, cytochrome c release and IP3R activity in muscular dystrophy. *Hum. Mol. Genet.* 22 4562–4578. 10.1093/hmg/ddt302 23804750

[B50] Gibbs E. M. Clarke N. F. Rose K. Oates E. C. Webster R. Feldman E. L. (2013). Neuromuscular junction abnormalities in DNM2-related centronuclear myopathy. *J. Mol. Med.* 91 727–737. 10.1007/s00109-013-0994-4 23338057

[B51] Goody M. F. Kelly M. W. Reynolds C. J. Khalil A. Crawford B. D. Henry C. A. (2012). NAD+ biosynthesis ameliorates a zebrafish model of muscular dystrophy. *PLoS Biol.* 10:e1001409. 10.1371/journal.pbio.1001409 23109907 PMC3479101

[B52] Granato M. van Eeden F. J. Schach U. Trowe T. Brand M. Furutani-Seiki M. (1996). Genes controlling and mediating locomotion behavior of the zebrafish embryo and larva. *Development* 123 399–413. 9007258 10.1242/dev.123.1.399

[B53] Gros-Louis F. Kriz J. Kabashi E. McDearmid J. Millecamps S. Urushitani M. (2008). Als2 mRNA splicing variants detected in KO mice rescue severe motor dysfunction phenotype in Als2 knock-down zebrafish. *Hum. Mol. Genet.* 17 2691–2702. 10.1093/hmg/ddn171 18558633

[B54] Gupta V. Kawahara G. Gundry S. R. Chen A. T. Lencer W. I. Zhou Y. (2011). The zebrafish dag1 mutant: A novel genetic model for dystroglycanopathies. *Hum. Mol. Genet.* 20, 1712–1725. 10.1093/hmg/ddr047 21296866 PMC3071669

[B55] Gupta V. A. Kawahara G. Myers J. A. Chen A. T. Hall T. E. Manzini M. C. (2012). A splice site mutation in laminin-α2 results in a severe muscular dystrophy and growth abnormalities in zebrafish. *PLoS One* 7:e43794. 10.1371/journal.pone.0043794 22952766 PMC3428294

[B56] Guyon J. R. Goswami J. Jun S. J. Thorne M. Howell M. Pusack T. (2009). Genetic isolation and characterization of a splicing mutant of zebrafish dystrophin. *Hum. Mol. Genet.* 18 202–211. 10.1093/hmg/ddn337 18957474 PMC2644651

[B57] Guyon J. R. Mosley A. N. Zhou Y. O’Brien K. F. Sheng X. Chiang K. (2003). The dystrophin associated protein complex in zebrafish. *Hum. Mol. Genet.* 12 601–615. 12620966

[B58] Guyon J. R. Steffen L. S. Howell M. H. Pusack T. J. Lawrence C. Kunkel L. M. (2007). Modeling human muscle disease in zebrafish. *Biochim. Biophys. Acta* 1772 205–215. 10.1016/j.bbadis.2006.07.003 16934958

[B59] Guyon J. Mosley A. Jun S. Montanaro F. Steffen L. Zhou Y. (2005). δ-Sarcoglycan is required for early zebrafish muscle organization. *Exp. Cell Res.* 304 105–115. 10.1016/j.yexcr.2004.10.032 15707578

[B60] Hall T. E. Bryson-Richardson R. J. Berger S. Jacoby A. S. Cole N. J. Hollway G. E. (2007). The zebrafish candyfloss mutant implicates extracellular matrix adhesion failure in laminin alpha2-deficient congenital muscular dystrophy. *Proc. Natl. Acad. Sci. U.S.A.* 104, 7092–7097. 10.1073/pnas.0700942104 17438294 PMC1855385

[B61] Hao L. T. Burghes A. H. Beattie C. E. (2011). Generation and characterization of a genetic zebrafish model of SMA carrying the human SMN2 gene. *Mol. Neurodegener.* 6:24. 10.1186/1750-1326-6-24 21443782 PMC3080329

[B62] Hao L. T. Wolman M. Granato M. Beattie C. E. (2012). Survival motor neuron affects plastin 3 protein levels leading to motor defects. *J. Neurosci.* 32 5074–5084. 10.1523/JNEUROSCI.5808-11.2012 22496553 PMC3355766

[B63] Herman I. Lopez M. A. Marafi D. Pehlivan D. Calame D. G. Abid F. (2021). Clinical exome sequencing in the diagnosis of pediatric neuromuscular disease. *Muscle Nerve* 63 304–310. 10.1002/mus.27112 33146414

[B64] Hewamadduma C. A. A. Grierson A. J. Ma T. P. Pan L. Moens C. B. Ingham P. W. (2013). Tardbpl splicing rescues motor neuron and axonal development in a mutant tardbp zebrafish. *Hum. Mol. Genet.* 22 2376–2386. 10.1093/hmg/ddt082 23427147 PMC3658164

[B65] Hirata H. Watanabe T. Hatakeyama J. Sprague S. M. Saint-Amant L. Nagashima A. (2007). Zebrafish relatively relaxed mutants have a ryanodine receptor defect, show slow swimming and provide a model of multi-minicore disease. *Development* 134 2771–2781. 10.1242/dev.004531 17596281

[B66] Howe K. Clark M. D. Torroja C. F. Torrance J. Berthelot C. Muffato M. (2013). The zebrafish reference genome sequence and its relationship to the human genome. *Nature* 496 498–503. 10.1038/nature12111 23594743 PMC3703927

[B67] Jagla K. Kalman B. Boudou T. Hénon S. Batonnet-Pichon S. (2017). Beyond mice: Emerging and transdisciplinary models for the study of early-onset myopathies. *Semin. Cell Dev. Biol.* 64 171–180. 10.1016/j.semcdb.2016.09.012 27670720

[B68] Jing L. Gordon L. R. Shtibin E. Granato M. (2010). Temporal and spatial requirements of unplugged/MuSK function during zebrafish neuromuscular development. *PLoS One* 5:e8843. 10.1371/journal.pone.0008843 20107509 PMC2809748

[B69] Jing L. Lefebvre J. L. Gordon L. R. Granato M. (2009). Wnt signals organize synaptic prepattern and axon guidance through the zebrafish unplugged/MuSK receptor. *Neuron* 61 721–733. 10.1016/j.neuron.2008.12.025 19285469 PMC2671566

[B70] Johnson N. M. Farr G. H. Maves L. (2013). The HDAC inhibitor TSA ameliorates a zebrafish model of duchenne muscular dystrophy. *PLoS Curr*. 5:ecurrents.md.8273cf41db10e2d15dd3ab827cb4b027. 10.1371/currents.md.8273cf41db10e2d15dd3ab827cb4b027 24459606 PMC3870918

[B71] Jones R. A. Harrison C. Eaton S. L. Llavero Hurtado M. Graham L. C. Alkhammash L. (2017). Cellular and molecular anatomy of the human neuromuscular junction. *Cell Rep.* 21 2348–2356. 10.1016/j.celrep.2017.11.008 29186674 PMC5723673

[B72] Joshi S. Virdi S. Etard C. Geisler R. Strähle U. (2018). Mutation of a serine near the catalytic site of the choline acetyltransferase a gene almost completely abolishes motility of the zebrafish embryo. *PLoS One* 13:e0207747. 10.1371/journal.pone.0207747 30458023 PMC6245786

[B73] Kabashi E. Bercier V. Lissouba A. Liao M. Brustein E. Rouleau G. A. (2011). FUS and TARDBP but not SOD1 interact in genetic models of amyotrophic lateral sclerosis. *PLoS Genet.* 7:e1002214. 10.1371/journal.pgen.1002214 21829392 PMC3150442

[B74] Kabashi E. Lin L. Tradewell M. L. Dion P. A. Bercier V. Bourgouin P. (2010). Gain and loss of function of ALS-related mutations of TARDBP (TDP-43) cause motor deficits in vivo. *Hum. Mol. Genet.* 19 671–683. 10.1093/hmg/ddp534 19959528

[B75] Kanning K. C. Kaplan A. Henderson C. E. (2010). Motor neuron diversity in development and disease. *Annu. Rev. Neurosci.* 33 409–440. 10.1146/annurev.neuro.051508.135722 20367447

[B76] Kawahara G. Guyon J. R. Nakamura Y. Kunkel L. M. (2010). Zebrafish models for human FKRP muscular dystrophies. *Hum. Mol. Genet.* 19 623–633. 10.1093/hmg/ddp528 19955119 PMC2807370

[B77] Kawahara G. Serafini P. R. Myers J. A. Alexander M. S. Kunkel L. M. (2011). Characterization of zebrafish dysferlin by morpholino knockdown. *Biochem. Biophys. Res. Commun.* 413 358–363. 10.1016/j.bbrc.2011.08.105 21893049 PMC4526276

[B78] Kimmel C. B. Ballard W. W. Kimmel S. R. Ullmann B. Schilling T. F. (1995). Stages of embryonic development of the zebrafish. *Dev. Dyn.* 203 253–310. 10.1002/aja.1002030302 8589427

[B79] Kohashi T. Oda Y. (2008). Initiation of Mauthner- or non-Mauthner-mediated fast escape evoked by different modes of sensory input. *J. Neurosci.* 28 10641–10653. 10.1523/JNEUROSCI.1435-08.2008 18923040 PMC6671347

[B80] Kummer T. T. Misgeld T. Sanes J. R. (2006). Assembly of the postsynaptic membrane at the neuromuscular junction: Paradigm lost. *Curr. Opin. Neurobiol.* 16 74–82. 10.1016/j.conb.2005.12.003 16386415

[B81] Laird A. S. Van Hoecke A. De Muynck L. Timmers M. Van den Bosch L. Van Damme P. (2010). Progranulin is neurotrophic in vivo and protects against a mutant TDP-43 induced axonopathy. *PLoS One* 5:e13368. 10.1371/journal.pone.0013368 20967127 PMC2954192

[B82] Lattante S. de Calbiac H. Le Ber I. Brice A. Ciura S. Kabashi E. (2015). Sqstm1 knock-down causes a locomotor phenotype ameliorated by rapamycin in a zebrafish model of ALS/FTLD. *Hum. Mol. Genet.* 24 1682–1690. 10.1093/hmg/ddu580 25410659

[B83] Lemmens R. Van Hoecke A. Hersmus N. Geelen V. D’Hollander I. Thijs V. (2007). Overexpression of mutant superoxide dismutase 1 causes a motor axonopathy in the zebrafish. *Hum. Mol. Genet.* 16 2359–2365. 10.1093/hmg/ddm193 17636250

[B84] Lescouzères L. Bomont P. (2020). E3 ubiquitin ligases in neurological diseases: Focus on gigaxonin and autophagy. *Front. Physiol.* 11:1022. 10.3389/fphys.2020.01022 33192535 PMC7642974

[B85] Lescouzères L. Hassen-Khodja C. Baudot A. Bordignon B. Bomont P. (Submitted). *A Multi-Disciplinary Screening Pipeline in Zebrafish Identifies Therapeutic Drugs for GAN.* 10.15252/emmm.202216267 PMC10331585 37144692

[B86] Li L. Zhou Q. Voss T. C. Quick K. L. LaBarbera D. V. (2016). High-throughput imaging: Focusing in on drug discovery in 3D. *Methods* 96 97–102. 10.1016/j.ymeth.2015.11.013 26608110 PMC4766031

[B87] Li M. Andersson-Lendahl M. Sejersen T. Arner A. (2013). Knockdown of desmin in zebrafish larvae affects interfilament spacing and mechanical properties of skeletal muscle. *J. Gen. Physiol.* 141 335–345. 10.1085/jgp.201210915 23440276 PMC3581687

[B88] Li M. Hromowyk K. J. Amacher S. L. Currie P. D. (2017). Muscular dystrophy modeling in zebrafish. *Methods Cell Biol.* 138 347–380. 10.1016/bs.mcb.2016.11.004 28129852

[B89] Lin P. Li J. Liu Q. Mao F. Li J. Qiu R. (2008). A missense mutation in SLC33A1, which encodes the acetyl-CoA transporter, causes autosomal-dominant spastic paraplegia (SPG42). *Am. J. Hum. Genet.* 83 752–759. 10.1016/j.ajhg.2008.11.003 19061983 PMC2668077

[B90] Lin Y.-Y. White R. J. Torelli S. Cirak S. Muntoni F. Stemple D. L. (2011). Zebrafish Fukutin family proteins link the unfolded protein response with dystroglycanopathies. *Hum. Mol. Genet.* 20 1763–1775. 10.1093/hmg/ddr059 21317159 PMC3071672

[B91] Luna V. M. Daikoku E. Ono F. (2015). “Slow” skeletal muscles across vertebrate species. *Cell Biosci.* 5:62. 10.1186/s13578-015-0054-6 26568818 PMC4644285

[B92] Lv X. Zhang R. Xu L. Wang G. Yan C. Lin P. (2022). Tcap deficiency in zebrafish leads to ROS production and mitophagy, and idebenone improves its phenotypes. *Front. Cell Dev. Biol.* 10:836464. 10.3389/fcell.2022.836464 35372370 PMC8964517

[B93] MacRae C. A. Peterson R. T. (2015). Zebrafish as tools for drug discovery. *Nat. Rev. Drug Discov.* 14 721–731. 10.1038/nrd4627 26361349

[B94] Manuel M. Zytnicki D. (2011). Alpha, beta and gamma motoneurons: Functional diversity in the motor system’s final pathway. *J. Integr. Neurosci.* 10 243–276. 10.1142/S0219635211002786 21960303

[B95] Manzini M. C. Tambunan D. E. Hill R. S. Yu T. W. Maynard T. M. Heinzen E. L. (2012). Exome sequencing and functional validation in zebrafish identify GTDC2 mutations as a cause of Walker-Warburg syndrome. *Am. J. Hum. Genet.* 91 541–547. 10.1016/j.ajhg.2012.07.009 22958903 PMC3512000

[B96] Mao F. Li Z. Zhao B. Lin P. Liu P. Zhai M. (2015). Identification and functional analysis of a SLC33A1: c.339T>G (p.Ser113Arg) variant in the original SPG42 family. *Hum. Mutat.* 36 240–249. 10.1002/humu.22732 25402622

[B97] Martin E. Schüle R. Smets K. Rastetter A. Boukhris A. Loureiro J. L. (2013). Loss of function of glucocerebrosidase GBA2 is responsible for motor neuron defects in hereditary spastic paraplegia. *Am. J. Hum. Genet.* 92 238–244. 10.1016/j.ajhg.2012.11.021 23332916 PMC3567271

[B98] Martin E. Yanicostas C. Rastetter A. Alavi Naini S. M. Maouedj A. Kabashi E. (2012). Spatacsin and spastizin act in the same pathway required for proper spinal motor neuron axon outgrowth in zebrafish. *Neurobiol. Dis.* 48 299–308. 10.1016/j.nbd.2012.07.003 22801083

[B99] McCammon J. M. Sive H. (2015). Challenges in understanding psychiatric disorders and developing therapeutics: A role for zebrafish. *Dis. Model Mech.* 8 647–656. 10.1242/dmm.019620 26092527 PMC4486859

[B100] McLean D. L. Fetcho J. R. (2008). Using imaging and genetics in zebrafish to study developing spinal circuits in vivo. *Dev. Neurobiol.* 68 817–834. 10.1002/dneu.20617 18383546 PMC3579555

[B101] McMacken G. Cox D. Roos A. Müller J. Whittaker R. Lochmüller H. (2018). The beta-adrenergic agonist salbutamol modulates neuromuscular junction formation in zebrafish models of human myasthenic syndromes. *Hum. Mol. Genet.* 27 1556–1564. 10.1093/hmg/ddy062 29462491 PMC5905648

[B102] McWhorter M. L. Monani U. R. Burghes A. H. M. Beattie C. E. (2003). Knockdown of the survival motor neuron (Smn) protein in zebrafish causes defects in motor axon outgrowth and pathfinding. *J. Cell Biol.* 162 919–931. 10.1083/jcb.200303168 12952942 PMC1761110

[B103] Menelaou E. McLean D. L. (2012). A gradient in endogenous rhythmicity and oscillatory drive matches recruitment order in an axial motor pool. *J. Neurosci.* 32 10925–10939. 10.1523/JNEUROSCI.1809-12.2012 22875927 PMC3428065

[B104] Mirat O. Sternberg J. R. Severi K. E. Wyart C. (2013). ZebraZoom: An automated program for high-throughput behavioral analysis and categorization. *Front. Neural Circuits* 7:107. 10.3389/fncir.2013.00107 23781175 PMC3679480

[B105] Müller J. S. Jepson C. D. Laval S. H. Bushby K. Straub V. Lochmüller H. (2010). Dok-7 promotes slow muscle integrity as well as neuromuscular junction formation in a zebrafish model of congenital myasthenic syndromes. *Hum. Mol. Genet.* 19 1726–1740. 10.1093/hmg/ddq049 20147321

[B106] Myers P. Z. (1985). Spinal motoneurons of the larval zebrafish. *J. Comp. Neurol.* 236 555–561. 10.1002/cne.902360411 4056102

[B107] Naef V. Mero S. Fichi G. D’Amore A. Ogi A. Gemignani F. (2019). Swimming in deep water: Zebrafish modeling of complicated forms of hereditary spastic paraplegia and spastic ataxia. *Front. Neurosci.* 13:1311. 10.3389/fnins.2019.01311 31920481 PMC6914767

[B108] Noldus L. P. Spink A. J. Tegelenbosch R. A. (2001). EthoVision: A versatile video tracking system for automation of behavioral experiments. *Behav. Res. Methods Instrum. Comput.* 33 398–414. 10.3758/bf03195394 11591072

[B109] O’Connor E. Phan V. Cordts I. Cairns G. Hettwer S. Cox D. (2018). MYO9A deficiency in motor neurons is associated with reduced neuromuscular agrin secretion. *Hum. Mol. Genet.* 27 1434–1446. 10.1093/hmg/ddy054 29462312 PMC5991207

[B110] O’Connor E. Töpf A. Müller J. S. Cox D. Evangelista T. Colomer J. (2016). Identification of mutations in the MYO9A gene in patients with congenital myasthenic syndrome. *Brain* 139 2143–2153. 10.1093/brain/aww130 27259756 PMC4958899

[B111] Oprişoreanu A.-M. Smith H. L. Arya S. Webster R. Zhong Z. Eaton-Hart C. (2019). Interaction of axonal chondrolectin with collagen XIXa1 is necessary for precise neuromuscular junction formation. *Cell Rep.* 29 1082–1098.e10. 10.1016/j.celrep.2019.09.033 31665626

[B112] Oprişoreanu A.-M. Smith H. L. Krix S. Chaytow H. Carragher N. O. Gillingwater T. H. (2021). Automated in vivo drug screen in zebrafish identifies synapse-stabilising drugs with relevance to spinal muscular atrophy. *Dis. Model Mech.* 14:dmm047761. 10.1242/dmm.047761 33973627 PMC8106959

[B113] Osborn D. P. S. Pond H. L. Mazaheri N. Dejardin J. Munn C. J. Mushref K. (2017). Mutations in INPP5K cause a form of congenital muscular dystrophy overlapping marinesco-sjögren syndrome and dystroglycanopathy. *Am. J. Hum. Genet.* 100 537–545. 10.1016/j.ajhg.2017.01.019 28190459 PMC5339112

[B114] Panzer J. A. Gibbs S. M. Dosch R. Wagner D. Mullins M. C. Granato M. (2005). Neuromuscular synaptogenesis in wild-type and mutant zebrafish. *Dev. Biol.* 285 340–357. 10.1016/j.ydbio.2005.06.027 16102744

[B115] Panzer J. A. Song Y. Balice-Gordon R. J. (2006). In vivo imaging of preferential motor axon outgrowth to and synaptogenesis at prepatterned acetylcholine receptor clusters in embryonic zebrafish skeletal muscle. *J. Neurosci.* 26 934–947. 10.1523/JNEUROSCI.3656-05.2006 16421313 PMC6675385

[B116] Pappalardo A. Pitto L. Fiorillo C. Alice Donati M. Bruno C. Santorelli F. M. (2013). Neuromuscular disorders in zebrafish: State of the art and future perspectives. *Neuromol. Med.* 15 405–419. 10.1007/s12017-013-8228-z 23584918

[B117] Parsons M. J. Campos I. Hirst E. M. A. Stemple D. L. (2002). Removal of dystroglycan causes severe muscular dystrophy in zebrafish embryos. *Development* 129 3505–3512. 10.1242/dev.129.14.3505 12091319

[B118] Patten S. A. Armstrong G. A. B. Lissouba A. Kabashi E. Parker J. A. Drapeau P. (2014). Fishing for causes and cures of motor neuron disorders. *Dis. Model Mech.* 7 799–809. 10.1242/dmm.015719 24973750 PMC4073270

[B119] Patton E. E. Zon L. I. Langenau D. M. (2021). Zebrafish disease models in drug discovery: From preclinical modelling to clinical trials. *Nat. Rev. Drug Discov.* 20 611–628. 10.1038/s41573-021-00210-8 34117457 PMC9210578

[B120] Peterson R. T. Fishman M. C. (2011). Designing zebrafish chemical screens. *Methods Cell Biol.* 105 525–541. 10.1016/B978-0-12-381320-6.00023-0 21951546

[B121] Pipis M. Rossor A. M. Laura M. Reilly M. M. (2019). Next-generation sequencing in Charcot-Marie-Tooth disease: Opportunities and challenges. *Nat. Rev. Neurol.* 15 644–656. 10.1038/s41582-019-0254-5 31582811

[B122] Postel R. Vakeel P. Topczewski J. Knöll R. Bakkers J. (2008). Zebrafish integrin-linked kinase is required in skeletal muscles for strengthening the integrin-ECM adhesion complex. *Dev. Biol.* 318 92–101. 10.1016/j.ydbio.2008.03.024 18436206

[B123] Radev Z. Hermel J.-M. Elipot Y. Bretaud S. Arnould S. Duchateau P. (2015). A TALEN-Exon skipping design for a bethlem myopathy model in zebrafish. *PLoS One* 10:e0133986. 10.1371/journal.pone.0133986 26221953 PMC4519248

[B124] Ramesh T. Lyon A. N. Pineda R. H. Wang C. Janssen P. M. L. Canan B. D. (2010). A genetic model of amyotrophic lateral sclerosis in zebrafish displays phenotypic hallmarks of motoneuron disease. *Dis. Model Mech.* 3 652–662. 10.1242/dmm.005538 20504969 PMC2931540

[B125] Reddy G. Desban L. Tanaka H. Roussel J. Mirat O. Wyart C. (2022). A lexical approach for identifying behavioural action sequences. *PLoS Comput. Biol.* 18:e1009672. 10.1371/journal.pcbi.1009672 35007275 PMC8782473

[B126] Rennekamp A. J. Peterson R. T. (2015). 15 years of zebrafish chemical screening. *Curr. Opin. Chem. Biol.* 24 58–70. 10.1016/j.cbpa.2014.10.025 25461724 PMC4339096

[B127] Robinson K. J. Yuan K. C. Don E. K. Hogan A. L. Winnick C. G. Tym M. C. (2019). Motor neuron abnormalities correlate with impaired movement in zebrafish that express mutant superoxide dismutase 1. *Zebrafish* 16 8–14. 10.1089/zeb.2018.1588 30300572 PMC6357263

[B128] Roscioli T. Kamsteeg E.-J. Buysse K. Maystadt I. van Reeuwijk J. van den Elzen C. (2012). Mutations in ISPD cause walker-warburg syndrome and defective glycosylation of α-dystroglycan. *Nat Genet* 44 581–585. 10.1038/ng.2253 22522421 PMC3378661

[B129] Ruparelia A. A. Oorschot V. Ramm G. Bryson-Richardson R. J. (2016). FLNC myofibrillar myopathy results from impaired autophagy and protein insufficiency. *Hum. Mol. Genet.* 25 2131–2142. 10.1093/hmg/ddw080 26969713

[B130] Ruparelia A. A. Zhao M. Currie P. D. Bryson-Richardson R. J. (2012). Characterization and investigation of zebrafish models of filamin-related myofibrillar myopathy. *Hum. Mol. Genet.* 21 4073–4083. 10.1093/hmg/dds231 22706277

[B131] Sakowski S. A. Lunn J. S. Busta A. S. Oh S. S. Zamora-Berridi G. Palmer M. (2012). Neuromuscular effects of G93A-SOD1 expression in zebrafish. *Mol. Neurodegener.* 7:44. 10.1186/1750-1326-7-44 22938571 PMC3506515

[B132] Sarparanta J. Jonson P. H. Golzio C. Sandell S. Luque H. Screen M. (2012). Mutations affecting the cytoplasmic functions of the co-chaperone DNAJB6 cause limb-girdle muscular dystrophy. *Nat. Genet.* 44 S1–S2. 10.1038/ng.1103 22366786 PMC3315599

[B133] Schmid B. Haass C. (2013). Genomic editing opens new avenues for zebrafish as a model for neurodegeneration. *J. Neurochem.* 127 461–470. 10.1111/jnc.12460 24117801

[B134] Schmid B. Hruscha A. Hogl S. Banzhaf-Strathmann J. Strecker K. van der Zee J. (2013). Loss of ALS-associated TDP-43 in zebrafish causes muscle degeneration, vascular dysfunction, and reduced motor neuron axon outgrowth. *Proc. Natl. Acad. Sci. U.S.A.* 110 4986–4991. 10.1073/pnas.1218311110 23457265 PMC3612625

[B135] See K. Yadav P. Giegerich M. Cheong P. S. Graf M. Vyas H. (2014). SMN deficiency alters Nrxn2 expression and splicing in zebrafish and mouse models of spinal muscular atrophy. *Hum. Mol. Genet.* 23 1754–1770. 10.1093/hmg/ddt567 24218366

[B136] Senderek J. Müller J. S. Dusl M. Strom T. M. Guergueltcheva V. Diepolder I. (2011). Hexosamine biosynthetic pathway mutations cause neuromuscular transmission defect. *Am. J. Hum. Genet.* 88 162–172. 10.1016/j.ajhg.2011.01.008 21310273 PMC3035713

[B137] Serafini P. R. Feyder M. J. Hightower R. M. Garcia-Perez D. Vieira N. M. Lek A. (2018). A limb-girdle muscular dystrophy 2I model of muscular dystrophy identifies corrective drug compounds for dystroglycanopathies. *JCI Insight* 3:e120493. 10.1172/jci.insight.120493 30232282 PMC6237228

[B138] Seth A. Stemple D. L. Barroso I. (2013). The emerging use of zebrafish to model metabolic disease. *Dis. Model Mech.* 6 1080–1088. 10.1242/dmm.011346 24046387 PMC3759328

[B139] Shaw M. P. Higginbottom A. McGown A. Castelli L. M. James E. Hautbergue G. M. (2018). Stable transgenic C9orf72 zebrafish model key aspects of the ALS/FTD phenotype and reveal novel pathological features. *Acta Neuropathol. Commun.* 6:125. 10.1186/s40478-018-0629-7 30454072 PMC6240957

[B140] Slater C. R. (2017). The structure of human neuromuscular junctions: Some unanswered molecular questions. *Int. J. Mol. Sci.* 18:E2183. 10.3390/ijms18102183 29048368 PMC5666864

[B141] Song Y. Wang M. Mao F. Shao M. Zhao B. Song Z. (2013). Knockdown of Pnpla6 protein results in motor neuron defects in zebrafish. *Dis. Model Mech.* 6 404–413. 10.1242/dmm.009688 22996643 PMC3597022

[B142] Swaminathan A. Bouffard M. Liao M. Ryan S. Callister J. B. Pickering-Brown S. M. (2018). Expression of C9orf72-related dipeptides impairs motor function in a vertebrate model. *Hum. Mol. Genet.* 27 1754–1762. 10.1093/hmg/ddy083 29528390 PMC5932562

[B143] Swenarchuk L. E. (2019). Nerve, muscle, and synaptogenesis. *Cells* 8:E1448. 10.3390/cells8111448 31744142 PMC6912269

[B144] Swinnen B. Bento-Abreu A. Gendron T. F. Boeynaems S. Bogaert E. Nuyts R. (2018). A zebrafish model for C9orf72 ALS reveals RNA toxicity as a pathogenic mechanism. *Acta Neuropathol.* 135 427–443. 10.1007/s00401-017-1796-5 29302778

[B145] Telfer W. R. Busta A. S. Bonnemann C. G. Feldman E. L. Dowling J. J. (2010). Zebrafish models of collagen VI-related myopathies. *Hum. Mol. Genet.* 19 2433–2444. 10.1093/hmg/ddq126 20338942 PMC2876888

[B146] Thornhill P. Bassett D. Lochmüller H. Bushby K. Straub V. (2008). Developmental defects in a zebrafish model for muscular dystrophies associated with the loss of fukutin-related protein (FKRP). *Brain* 131 1551–1561. 10.1093/brain/awn078 18477595

[B147] Valdmanis P. N. Meijer I. A. Reynolds A. Lei A. MacLeod P. Schlesinger D. (2007). Mutations in the KIAA0196 gene at the SPG8 locus cause hereditary spastic paraplegia. *Am. J. Hum. Genet.* 80 152–161. 10.1086/510782 17160902 PMC1785307

[B148] van der Meer D. L. M. Marques I. J. Leito J. T. D. Besser J. Bakkers J. Schoonheere E. (2006). Zebrafish cypher is important for somite formation and heart development. *Dev. Biol.* 299 356–372. 10.1016/j.ydbio.2006.07.032 16982050

[B149] Vaz R. Hofmeister W. Lindstrand A. (2019). zebrafish models of neurodevelopmental disorders: Limitations and benefits of current tools and techniques. *Int. J. Mol. Sci.* 20:E1296. 10.3390/ijms20061296 30875831 PMC6471844

[B150] Vettori A. Bergamin G. Moro E. Vazza G. Polo G. Tiso N. (2011). Developmental defects and neuromuscular alterations due to mitofusin 2 gene (MFN2) silencing in zebrafish: A new model for Charcot-Marie-Tooth type 2A neuropathy. *Neuromuscul. Disord.* 21 58–67. 10.1016/j.nmd.2010.09.002 20951042

[B151] Vissing J. Johnson K. Töpf A. Nafissi S. Díaz-Manera J. French V. M. (2019). POPDC3 gene variants associate with a new form of limb girdle muscular dystrophy. *Ann. Neurol.* 86 832–843. 10.1002/ana.25620 31610034

[B152] Westerfield M. McMurray J. V. Eisen J. S. (1986). Identified motoneurons and their innervation of axial muscles in the zebrafish. *J. Neurosci.* 6 2267–2277. 3746409 10.1523/JNEUROSCI.06-08-02267.1986 PMC6568761

[B153] Westra D. Schouten M. I. Stunnenberg B. C. Kusters B. Saris C. G. J. Erasmus C. E. (2019). Panel-based exome sequencing for neuromuscular disorders as a diagnostic service. *J. Neuromuscul. Dis.* 6 241–258. 10.3233/JND-180376 31127727

[B154] Weterman M. A. J. Sorrentino V. Kasher P. R. Jakobs M. E. van Engelen B. G. M. Fluiter K. (2012). A frameshift mutation in LRSAM1 is responsible for a dominant hereditary polyneuropathy. *Hum. Mol. Genet.* 21 358–370. 10.1093/hmg/ddr471 22012984 PMC3276280

[B155] Widrick J. J. Alexander M. S. Sanchez B. Gibbs D. E. Kawahara G. Beggs A. H. (2016). Muscle dysfunction in a zebrafish model of Duchenne muscular dystrophy. *Physiol. Genomics* 48 850–860. 10.1152/physiolgenomics.00088.2016 27764767 PMC6223571

[B156] Winkler C. Eggert C. Gradl D. Meister G. Giegerich M. Wedlich D. (2005). Reduced U snRNP assembly causes motor axon degeneration in an animal model for spinal muscular atrophy. *Genes Dev.* 19 2320–2330. 10.1101/gad.342005 16204184 PMC1240041

[B157] Wolman M. Granato M. (2012). Behavioral genetics in larval zebrafish: Learning from the young. *Dev. Neurobiol.* 72 366–372. 10.1002/dneu.20872 22328273 PMC6430578

[B158] Wood J. D. Landers J. A. Bingley M. McDermott C. J. Thomas-McArthur V. Gleadall L. J. (2006). The microtubule-severing protein Spastin is essential for axon outgrowth in the zebrafish embryo. *Hum. Mol. Genet.* 15 2763–2771. 10.1093/hmg/ddl212 16893913

[B159] Wu H. Xiong W. C. Mei L. (2010). To build a synapse: Signaling pathways in neuromuscular junction assembly. *Development* 137 1017–1033. 10.1242/dev.038711 20215342 PMC2835321

[B160] Xu L. Geng H. Lv X. Wang G. Yan C. Zhang D. (2022). A female carrier of spinal and bulbar muscular atrophy diagnosed with DNAJB6-related distal myopathy. *J. Hum. Genet*. 67 441–444. 10.1038/s10038-022-01022-3 35165376

[B161] Yeh T.-H. Liu H.-F. Li Y.-W. Lu C.-S. Shih H.-Y. Chiu C.-C. (2018). C9orf72 is essential for neurodevelopment and motility mediated by Cyclin G1. *Exp. Neurol.* 304 114–124. 10.1016/j.expneurol.2018.03.002 29522758

[B162] Zhang R. Yang J. Zhu J. Xu X. (2009). Depletion of zebrafish Tcap leads to muscular dystrophy via disrupting sarcomere–membrane interaction, not sarcomere assembly. *Hum. Mol. Genet.* 18 4130–4140. 10.1093/hmg/ddp362 19679566 PMC2758143

[B163] Zhao M. Smith L. Volpatti J. Fabian L. Dowling J. J. (2019). Insights into wild-type dynamin 2 and the consequences of DNM2 mutations from transgenic zebrafish. *Hum. Mol. Genet.* 28 4186–4196. 10.1093/hmg/ddz260 31691805

[B164] Zhong Z. Ohnmacht J. Reimer M. M. Bach I. Becker T. Becker C. G. (2012). Chondrolectin mediates growth cone interactions of motor axons with an intermediate target. *J. Neurosci.* 32 4426–4439. 10.1523/JNEUROSCI.5179-11.2012 22457492 PMC6622066

[B165] Zhou Y. Cattley R. T. Cario C. L. Bai Q. Burton E. A. (2014). Quantification of larval zebrafish motor function in multiwell plates using open-source MATLAB applications. *Nat. Protoc.* 9 1533–1548. 10.1038/nprot.2014.094 24901738 PMC4169233

[B166] Zivony-Elboum Y. Westbroek W. Kfir N. Savitzki D. Shoval Y. Bloom A. (2012). A founder mutation in Vps37A causes autosomal recessive complex hereditary spastic paraparesis. *J. Med. Genet.* 49 462–472. 10.1136/jmedgenet-2012-100742 22717650

[B167] Zon L. I. Peterson R. T. (2005). In vivo drug discovery in the zebrafish. *Nat. Rev. Drug Discov.* 4 35–44. 10.1038/nrd1606 15688071

[B168] Zulian A. Rizzo E. Schiavone M. Palma E. Tagliavini F. Blaauw B. (2014). NIM811, a cyclophilin inhibitor without immunosuppressive activity, is beneficial in collagen VI congenital muscular dystrophy models. *Hum. Mol. Genet.* 23 5353–5363. 10.1093/hmg/ddu254 24852368

