# Supplementary material for: Development of a high-throughput tailored imaging method in zebrafish to understand and treat neuromuscular diseases
Source: Front Mol Neurosci. 2022 Sep 20;15:956582. doi: 10.3389/fnmol.2022.956582 (PMC9530744; doi:10.3389/fnmol.2022.956582)
Supplement: Supplementary file 1 [file Image_1.pdf]

Supplementary material

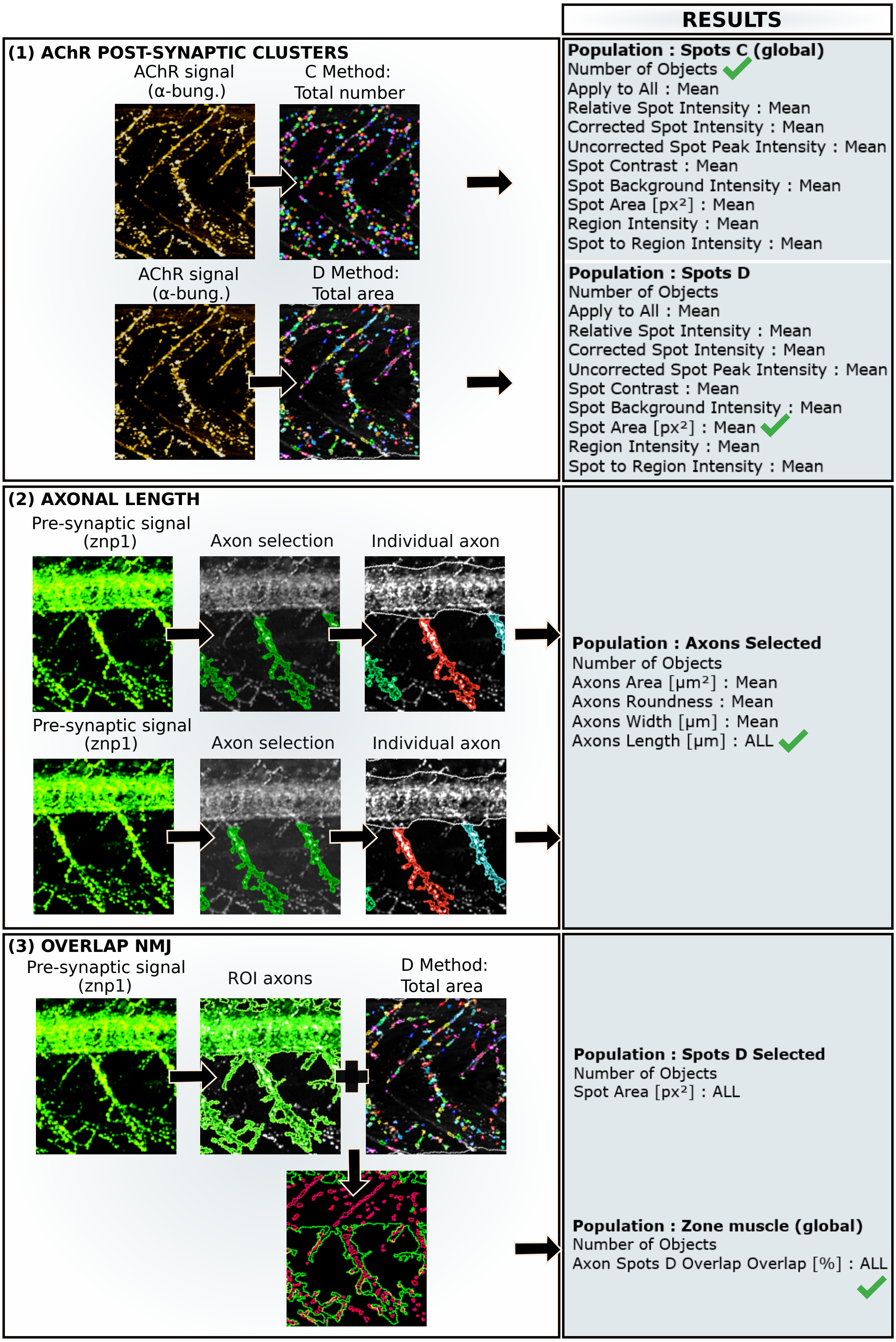

Supplementary Figure 1. Representative high-magnification images of the different filters for the segmentation of (1) AChR post-synaptic clusters from α-bungarotoxin staining (α-bung.), (2) individual axon from the znp1 staining, and (3) overlap NMJ resulting from the merged between (1) and (2) pictures. Each of these three modules (raw data and processed images) allows the extraction of quantitative parameters (right panel, results from Analysis sequence of the Harmony Software). The green sign indicates the specific parameters we used in the study.
